# Deep learning approach for predicting functional Z-DNA regions using omics data

**DOI:** 10.1038/s41598-020-76203-1

**Published:** 2020-11-05

**Authors:** Nazar Beknazarov, Seungmin Jin, Maria Poptsova

**Affiliations:** Laboratory of Bioinformatics, Faculty of Computer Science, National Research University Higher School of Economics, 11 Pokrovsky boulvar, Moscow, Russia 101000

**Keywords:** Epigenomics, Computational biology and bioinformatics, Computational models, Data integration, Data processing, Machine learning

## Abstract

Computational methods to predict Z-DNA regions are in high demand to understand the functional role of Z-DNA. The previous state-of-the-art method Z-Hunt is based on statistical mechanical and energy considerations about B- to Z-DNA transition using sequence information. Z-DNA CHiP-seq experiment results showed little overlap with Z-Hunt predictions implying that sequence information only is not sufficient to explain emergence of Z-DNA at different genomic locations. Adding epigenetic and other functional genomic mark-ups to DNA sequence level can help revealing the functional Z-DNA sites. Here we take advantage of the deep learning approach that can analyze and extract information from large volumes of molecular biology data. We developed a machine learning approach DeepZ that aggregates information from genome-wide maps of epigenetic markers, transcription factor and RNA polymerase binding sites, and chromosome accessibility maps. With the developed model we not only verify the experimental Z-DNA predictions, but also generate the whole-genome annotation, introducing new possible Z-DNA regions, which have not yet been found in experiments and can be of interest to the researchers from various fields.

## Introduction

After discovery of a standard form of DNA, which is the canonical right-handed B-form, or B-DNA^1^, other DNA configurations were found to exist. One of them is a left-handed DNA, termed as Z-DNA, discovered unexpectedly during solving the structure of a crystalline fragment of double-helical DNA^2^. Investigation of the crystalline structure revealed characteristic properties of nucleotides in Z-DNA—a regular alternation of *syn* and *anti* base conformations along each strand of the helix. Early experimental evidence confirmed presence of Z-DNA regions in viruses^3^, bacteria^4^ and mammals^5^. Later Z-DNA was also found in yeast^6^, fly^7^, and humans^8,9^.

Z-DNA has diverse functional roles, and many of them are yet to be discovered. Z-DNA located in promoter regions may work as a regulator of transcription. Association of promoter Z-DNA with transcription was found for C-MYC gene^10^, corticotropin-releasing hormone gene^11^, and heme oxygenase-1 gene (HO-1)^12^. Z-DNA was confirmed to act as a repressor in the promoter of ADAM-12^13^, known to be overexpressed in many human cancers. Mammalian protein DAI, a DNA-dependent activator of the innate immune response, senses cytosolic DNA by using two Z-DNA binding domains^14^. Proteins that are known to bind specifically to Z-DNA, their properties and potential biological functions are reviewed in^15^.

Z-DNA may promote homologous recombination by increasing frequencies up to twofold^16^. Z-DNA causes genome instability such as large-scale deletions in mammalian cells and small deletions in bacteria^17^. Z-DNA-forming regions are found in Alu retrotransposons, and one of Alu Z-DNA sites overlap with the binding site of signal recognition particles in the right arm of Alu^18^. Alu Z-DNA binding with ADAR diminishes retrotransposition activities, which likely played an essential role in evolution of primate genomes. Also, Z-DNA sites were shown to act as chromatin remodelers^19^.

Z-DNA was found to be associated with different diseases (see^18,20^ for reviews). Z-DNA was detected in the hippocampal region of brain samples severely affected by Alzheimer's disease^21^. ADAM proteins, that contain Z-DNA in the promoter region, are associated with various metabolic and inflammatory diseases, such as diabetes, sepsis, Alzheimer's disease and rheumatoid arthritis^22^. The variants of ADAR with either absent or mutated Zα domain affect interferon responses and are associated with rare Mendelian diseases: Dyschromatosis Symmetrica Hereditaria, Aicardi-Goutières syndrome, and Bilateral Striatal Necrosis/Dystonia^23^. Overexpression of ADAR suppresses inner immune response by inhibiting interferon production and leads to tumor progression^24^.

Z-DNA formation occurs in regions of negatively supercoiled DNA^25^, which can be generated upstream of polymerases^26^. Negatively supercoiled DNA can also be formed as a result of action of chromatin remodelers that removes nucleosomes in order to provide for promoter and enhancer accessibility^27^. Z-DNA can possibly act as a nucleosome barrier^28,29^, but the overall role of Z-DNA in shaping chromatin structure is yet to be determined. It was shown that certain DNA modifications (5mC, 5fC, 5cC, 8-oxo, 8-nitro, 7-methyl-purines, 2′ OmR) affect Z-DNA transitions^30^.

The experiments for detection of Z-DNA structure have many biases (see^30^ for a summary), that is why currently there are only few whole genome maps are available. The first Z-DNA map of the human genome was generated by using Zα domain of the double-stranded RNA editing enzyme ADAR^31^. 186 Z-DNA hotspots were found, among which 46 hotspots were located in centromeres of 13 human chromosomes. Unexpectedly only 2 hotspots were located near transcription start sites.

The first ChIP-Seq experiment for detection of Z-DNA regions was published in^9^. To generate a genome-wide map of Z-DNA sites the authors used Zaa protein with two Z-DNA-binding domains. The resulting map contained 391 regions with the majority of the Z-DNA located in promoter areas. Also, the detected Z-DNA regions showed enrichment in active histone marks H3K4me3 and H3K9ac, suggesting association of Z-DNA sites with active transcription. Additional analysis of RNA polymerase II ChIP-Seq data revealed that almost 60% of Z-DNA regions overlapped with RNA polymerase II peaks.

Two similar techniques of mapping non-B DNA structures became recently available. The first is based on potassium permanganate footprinting^32^ and the second on kethoxal-assisted single-stranded DNA sequencing^33^. Both methods first generate a map of single-stranded DNA and then ssDNA regions are superimposed with computationally predicted non-B DNA structures, including Z-DNA. Both methods revealed a high potential of human genome to form non-B DNA structures however the limitation of this approach is an algorithmic prediction of Z-DNA forming regions.

Z-DNA’s properties and statistical mechanical considerations about transition of the right-handed to the left-handed form were put at the basis of Z-Hunt, the first computer program for predicting regions of Z-DNA in long DNA sequences^34,35^. Z-Hunt algorithm considers the stability of Z-DNA as the difference in free energy between the right-handed B- and left-handed Z-DNA and employs statistical mechanical approach for B- to Z-DNA transition induced by negative supercoiling. Energetic parameters for dinucleotides associated with B to Z transition were taken from experiments^34^. The analysis of the human DNA 1 Mb fragment containing 137 genes revealed 329 potential Z-DNA-forming sequences, many of them found near transcription initiation sites^35^.

Computer methods for Z-DNA sites prediction are based on the assumption that Z-DNA regions are formed at sites with alternating purines and pyrimidines. Analysis of the detected Z-DNA regions revealed that only 40% of the sequences have alternating purine/pyrimidine pattern, suggesting that this is not the only major factor required to form Z-DNA. Comparison of the detected 391 regions with the set of 186 identified in^8^ showed little overlap—only 6 Z-DNA regions in common. This finding reveals difficulties in detection of Z-DNA regions, both computationally and experimentally.

Advances in machine learning, and especially in deep learning made it possible to create machine learning models that outperformed many existing computer models. The success of deep learning models in prediction of DNA functional elements can be explained by the aggregation of many factors and features into the training set so that neural network can take advantage of the maximum information available. The deep learning applications include prediction of gene expression^36^ and differential gene expression from histone modification signals^37^, histone modifications from sequence information and chromatin accessibility data^38^, protein-RNA binding preferences from sequence and RNA-secondary structure information^39^, promoters and enhancers from histone modification and TF binding ChIP-seq, DNase-seq, FAIRE-seq, and ChIA-PET data^40^.

The difficulty in detecting non-B DNA structures in general, and Z-DNA in particular, is that they are dynamically formed, perform they function and then disassemble. That is why it is difficult to perform genome-wide experiments for their detection, and the existing experiments are limited to the subset of DNA structures that were active at the time of experiment. Originally, the computational method Z-Hunt was based on sequence information only. Nowadays we have many omics data that could help to decipher the genome regulatory code, and specifically the regulatory code of Z-DNA. Here we, for the first time, present deep learning model to predict Z-DNA regions incorporating information about sequence, epigenetic code, chromatin accessibility, and transcription factor and RNA polymerase binding sites.

## Results

### Choosing the best model

For Z-DNA recognition task, we tested different machine learning models, comprising three types of deep learning approaches: convolution neural networks (CNN), recurrent neural networks (RNN), and hybrid CNN-RNN models. All three neural network architectures have been successfully applied to various tasks, specifically for recognition of functional genomic elements. However it is difficult to predict in advance, which architecture will be best suited for the task of Z-DNA recognition, that is why we tested many different models combining different number of machine learning blocks in order to choose the best model, which will be used for whole-genome annotations.

**Figure 1:**
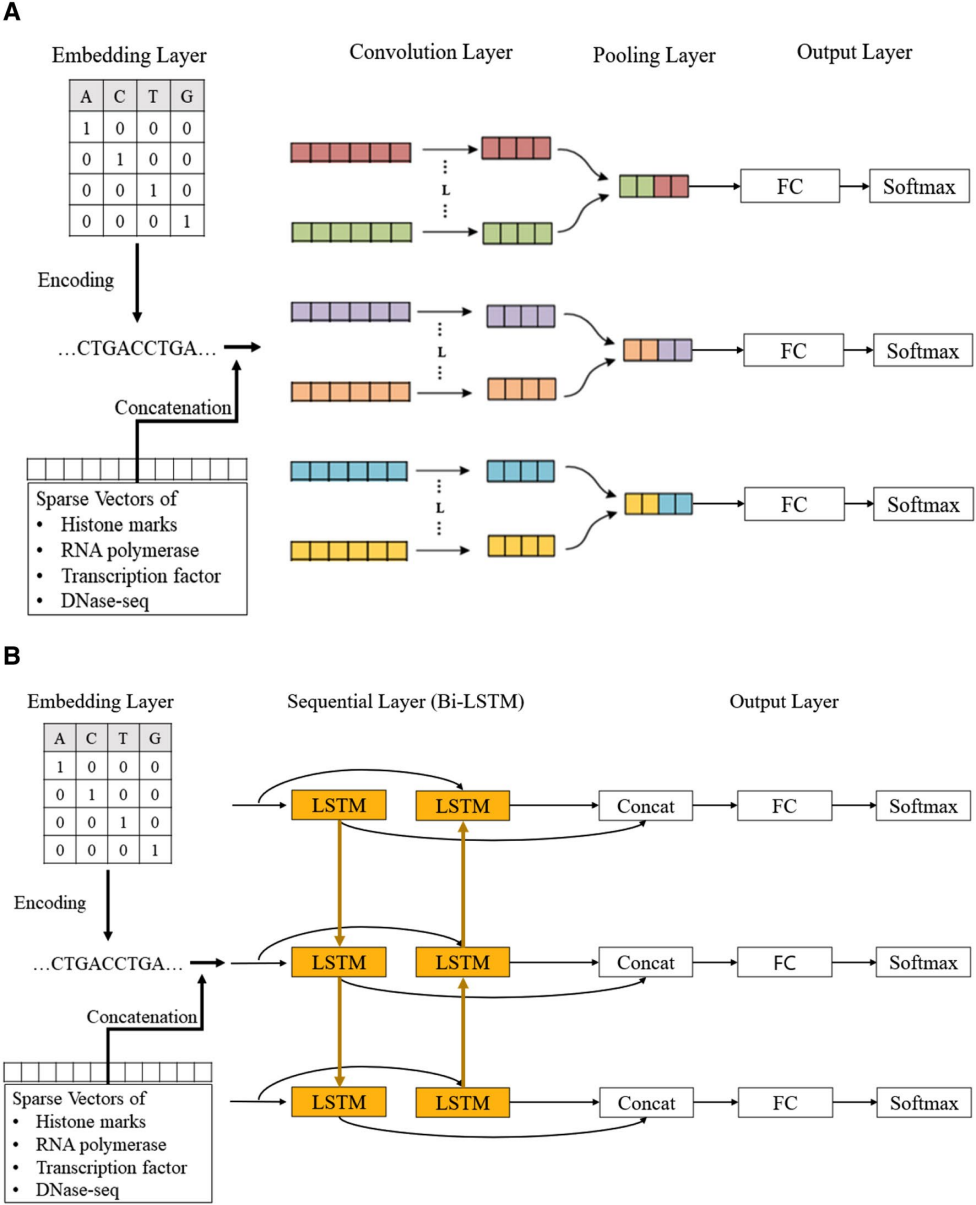
General schema of deep learning models for Z-DNA prediction. (**A**) CNN based deep models architectures. Convolution layers consist of 1 dimensional, and the result passed to FC layer after the max pooling process. (**B**) RNN based deep models architecture for Z-DNA prediction. The second LSTM cell takes reversed order of data then concat the result with the first LSTM cell with the original order to improve the performance.

**Figure 2:**
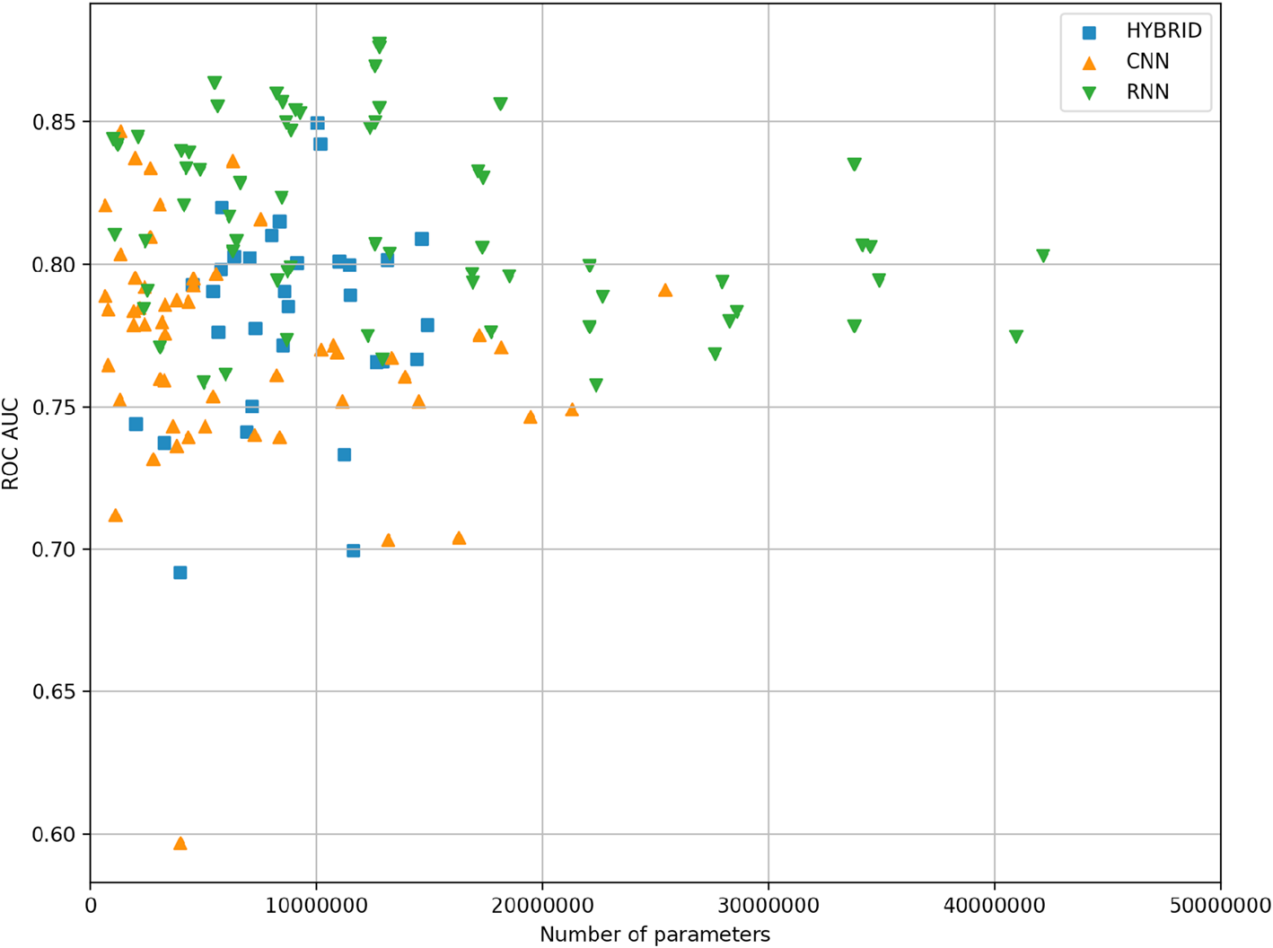
Comparison of 151 deep learning model performances on the test set. Every point represents one model.

The general schemes for CNN and RNN blocks are presented in Fig. 1. Different deep learning models were constructed by combining different numbers of blocks and layers inside one block. We totally trained and tested 151 models from which 54 were constructed using CNN-based architecture, 65—RNN-based architectures, and 32—hybrid CNN-RNN architectures. The results of different model performance are presented in Fig. 2. The best model is selected by two metrics: the area under the receiver operating characteristic curve (ROC AUC) (it provides an aggregate measure of performance across all possible classification thresholds) and F1 score (it is the harmonic mean of precision and recall, and it gives a better measure of the incorrectly classified cases than the accuracy), since these two metrics are resistant to class imbalances. The best performing deep learning model appeared to be RNN with 86.6% ROC AUC and 40.1% F1 score on the test set. The architecture of the best RNN model consists of two bidirectional long short-term memory (LSTM) networks followed by two fully connected (FC) networks with two dropout layers and sigmoid activation (see “Methods” section, Fig. 1B).

**Figure 3:**
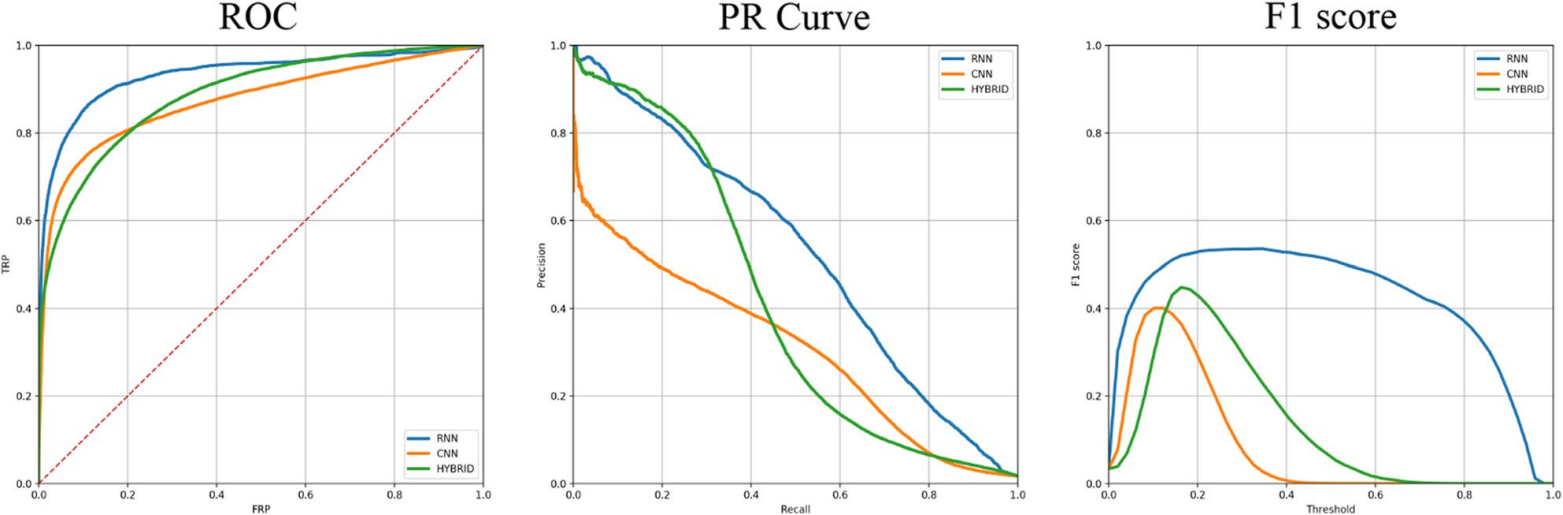
Comparison of best models from each class: CNN, RNN and hybrid CNN-RNN.

For comparison, the best CNN model consisted of 1 CNN layer, and 1 FC layer with 3 kernels and achieved 84.7% ROC AUC and 38.2% F1 score. The best hybrid CNN-RNN model consisted of two CNN layers followed by bidirectional LSTM with two FC layers, two dropouts and sigmoid activation. This architecture reached 85% ROC AUC and 39.1% F1 score. Best model comparison from each of the class—CNN, RNN and hybrid CNN-RNN is presented in Fig. 3.

For further experiments we chose the model based on the best RNN architecture and hereinafter referred as DeepZ. The model was trained on two Z-DNA datasets. The first data set is composed of Z-DNA regions detected by ChIP-Seq experiment of Shin et al.^9^ referred to hereafter as Shin data set. The second data set is composed of the combined data sets of experiments of Kouzine et al.^32,33^ and Wu et al.^33^ referred to hereafter as Kouzine-Wu data set. Kouzine-Wu data set is composed of Z-DNA regions inferred with the mixed experimental and computer approach: experimentally detected single-stranded DNA regions (ssDNA) are overlapped with computer predictions by Z-Hunt. We filtered all data sets from ENCODE blacklist regions^41^. Additionally to the sequence data as an input we used about 30,000 ChIP-Seq experiments on histone modifications, transcriptions factor and RNA polymerase binding sites, and chromatin state, various DNA methylation and DNA methylation variants maps, and B-Z transition energies of dinucleotide pairs (see “Methods” section).

### Comparison of DeepZ and Z-Hunt

**Figure 4:**
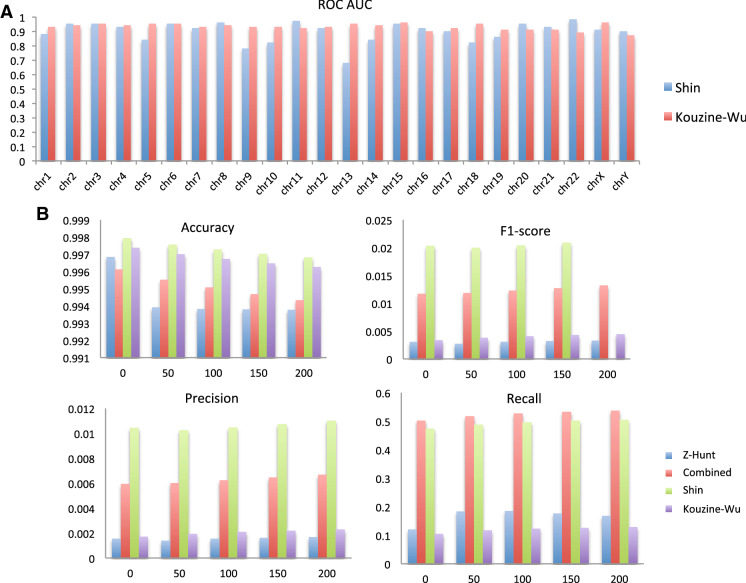
DeepZ model performance. (**A**) Per chromosome comparison of DeepZ best model performance trained on Shin and Kouzine-Wu data sets. (**B**) Comparizon of DeepZ and Z-Hunt models in predicting the Shin data set.

We trained DeepZ on two datasets of Shin and Kouzine-Wu and performance of deepZ model for every chromosome is depicted in Fig. 4A. The lowest performance was found for chromosomes 13 and 9 for DeepZ trained on Shin data set and for chromosomes 22 and Y for DeepZ trained on Kouzine-Wu data set; while the best performance was achieved for chromosome 11 and 22 (DeepZ on Shin data set) and for chromosomes 15 and X (DeepZ on Kouzine-Wu data set). For the remaining chromosomes the ROC AUC metric behavior is more uniform for all chromosomes having the value of more than 80%.

Comparison of ChIP-seq Z-DNA prediction by the DeepZ model with the current computer prediction method Z-Hunt is presented in Fig. 4B. We tested several conditions whether method predicts at least one Z-DNA nucleotide within a region of experimentally determined Z-DNA or Z-DNA is located at some distance from the determined nucleotides. The rationale behind the last assumption is that epigenetic and regulatory code used to train the deep learning model might help to determine not the exact sites but an area where Z-DNA region can be located. We considered different regions of 50, 100, 150, and 200 bp around the selected nucleotide. DeepZ outperforms Z-Hunt according to every measured metric, but especially in F1 score and precision, that are the most important performance metrics for our task.

### Whole-genome annotation with Z-DNA regions

We developed a whole-genome annotation procedure in which DeepZ assigns a probability to every nucleotide to be in a Z-DNA region. Then we assembled regions based on the sequence of nucleotides with a probability more than a designated threshold (see “Methods” section).

Because two training data sets are of different nature—the Shin data set is small but purely experimental (380 regions) while the Kouzine-Wu data set is large (47,774) but is a mixture of experimental and computer approaches—we decided not to combine these two data sets but to train two DeepZ models on two different data sets separately, generate two annotations made independently by two models, and also make the third combined annotation as a union of the two. Three whole-genome annotations of Z-DNA regions can be found in Supplementary Files S1–S3. The Z-DNA maps can be uploaded as a UCSC genome browser custom track. DeepZ trained on Shin data set predicted 43,873 regions, DeepZ trained on Kouzine-Wu data set predicted 46,398 regions, and the overlap between the two comprised 19,201 regions (44% for Shin and 41% for Kouzine-Wu data set). The combined data set resulted in 70,282 (Z-DNA regions closer than 10 bp were combined).

**Figure 5:**
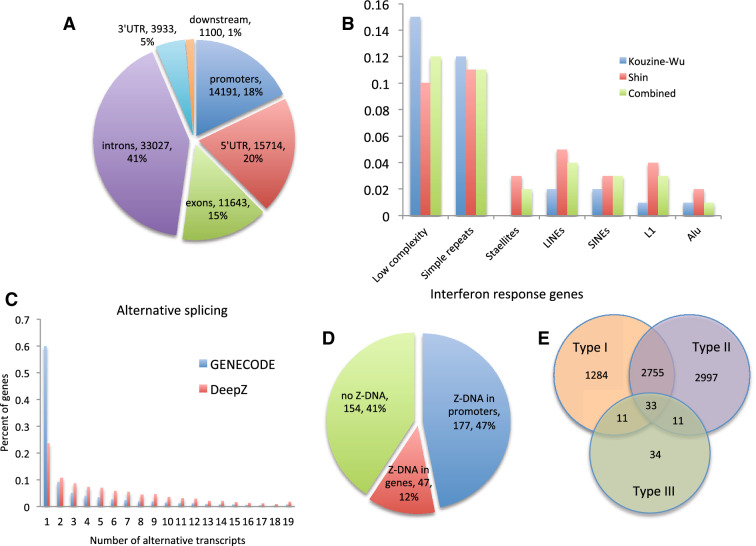
(**A**) Distribution of combined DeepZ predicted Z-DNA regions over genomic regions. (**B**) Distribution of Shin, Kouzine-Wu and combined DeepZ predicted Z-DNA regions over genomic repeats. (**C**) Comparison of GENCODE genes having various number of alternative transcripts over DeepZ predicted genes with Z-DNA regions. (**D**) Distributions of interferon response genes with Z-DNA regions either in gene bodies or promoters. (**E**) Venn diagram for the number of genes participating in interferon response of different types (generated by Inteferome DB^42^).

The distribution of Z-DNA predicted regions of the combined data set over gene regions is depicted in Fig. 5A, and distributions of Z-DNA regions predicted based on Shin and Kouzine-Wu data sets are given in Supplementary Fig. S1. Qualitatively genomic distributions of both Shin and Kouzine-Wu DeepZ predictions are the same with 63–66% of the regions falling inside the genes with the remaining 37–33% being in the intergenic regions. If we take only gene areas with promoters (upstream 1000 bp) and 1000 bp downstream regions, then DeepZ predictions on Shin data set are more enriched in 5′ UTR (28% over 19%) and promoters (25% over 17%) compared to DeepZ predictions on Kouzine-Wu data sets. The full list of DeepZ-predicted genes harboring Z-DNA in promoters and genes is provided in Supplementary Files S4 and S5.

The distribution of three DeepZ-generated Z-DNA maps over repeats (Fig. 5B) showed that 24–27% of predictions fall into simple repeats, satellites and low complexity regions, which is explainable since Z-DNA regions are often formed by purine-pyrimidine repeats. The percentage of Alu and L1, and SINEs and LINEs in general are low (1–3%), which is explained by the data processing pipelines that remove repeats before peak calling.

We found genes with predicted Z-DNA regions are enriched in alternative splicing events (Fig. 5C, p < e−10, Mann–Whitney U test). Distribution of GENCODE genes with different number of transcripts (the average number of transcripts per gene is 3.8) and genes with DeepZ-predicted Z-DNA regions (the average number of transcripts per gene is 7.35) are shown in Fig. 5C.

Because of the association ADAR variants with interferon responses and Mendelian diseases, we tested, how many of the genes with DeepZ predicted Z-DNA in genes or promoters are Interferon response genes (IRGs). Out of 378 reported in^43^ as genes participating in type I interferon response 177 have predicted Z-DNA regions in promoters and 224 in both gene bodies and promoter areas (Fig. 5D and Supplementary Table S1). Analysis of all types of IRGs revealed that 53% (7,125 out of 13,444) of genes with promoter Z-DNA are found in the Interferome database^42^, and 51% (8865 out of 17,370) of genes with Z-DNA in promoters or gene bodies (Supplementary Table S1). The distribution of genes over interferon types is given in Fig. 5E.

**Figure 6:**
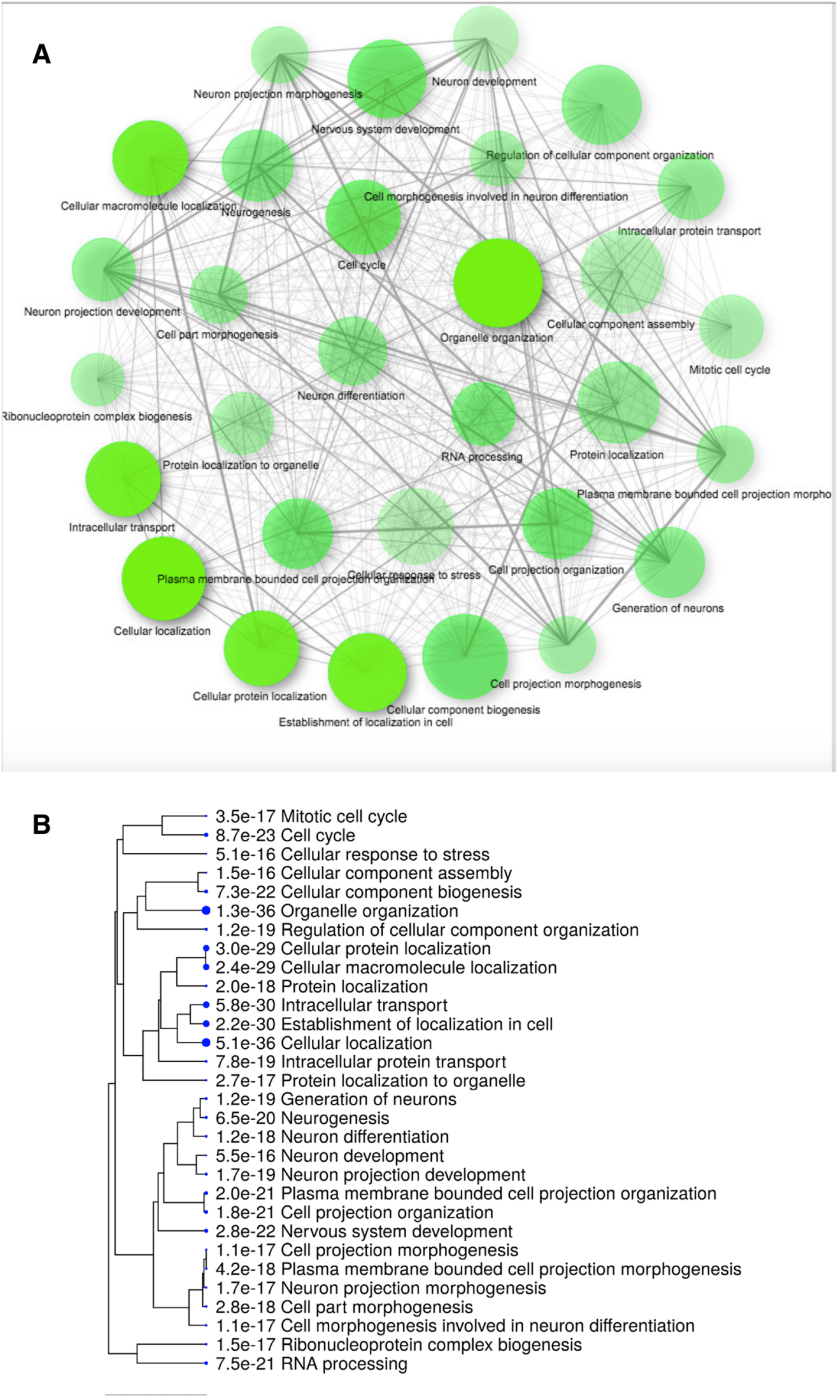
GO enrichment analysis for genes with predicted Z-DNA regions in promoter regions (see Supplementary Table S2 for a list of genes in each category). (**A**) Network representation (generated with ShinyGO^44^). (**B**) Tree representation (generated with ShinyGO^44^). The corresponding Figure for GO enrichment analysis for genes with predicted Z-DNA regions in gene bodies can be found in Supplementary Fig. S2.

GO-enrichment analysis for genes with predicted Z-DNA regions in promoter regions (Fig. 6) and gene bodies (Supplementary Fig. S2) revealed functional categories associated with cellular localization, cellular response to stress, neurogenesis, and others. The full list of the significant functional categories with the list of corresponding genes harboring Z-DNA regions can be found in Supplementary Tables S2.

**Figure 7:**
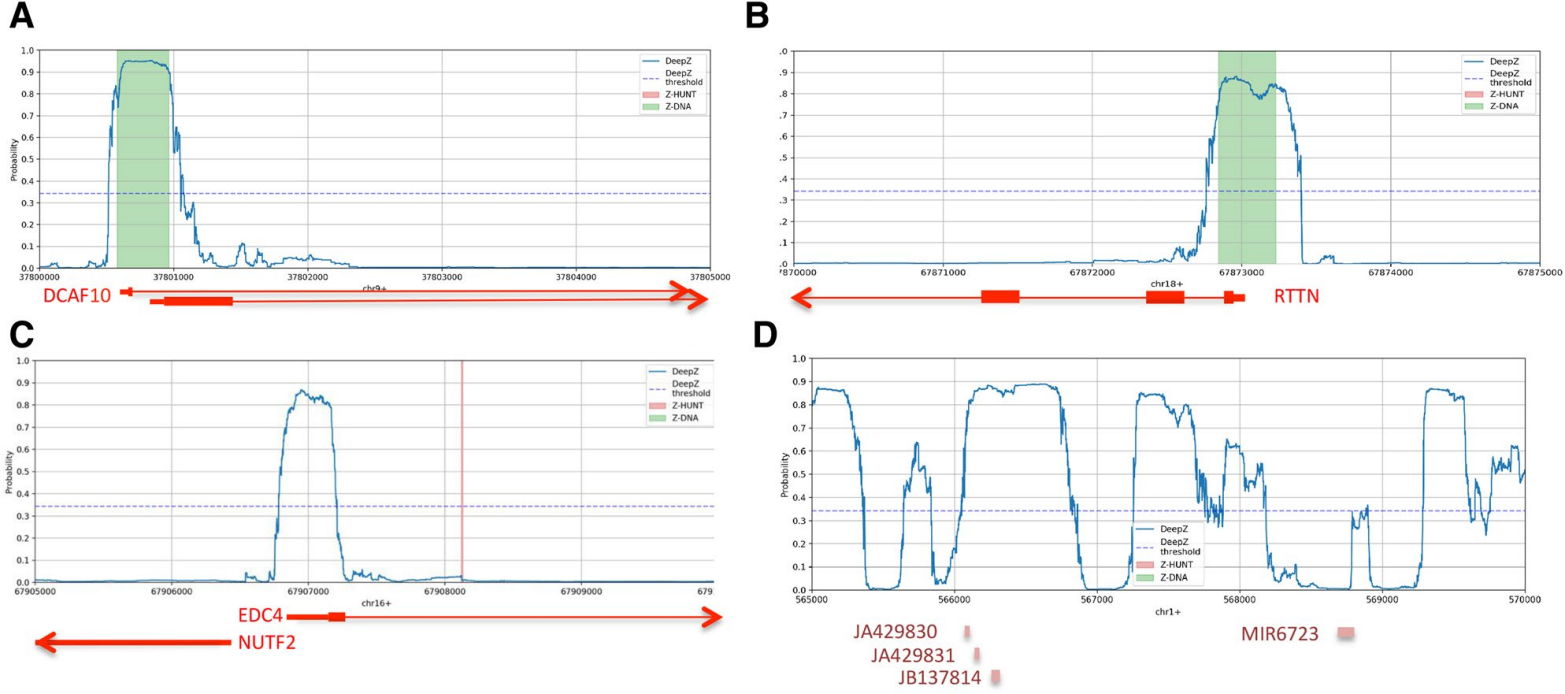
DeepZ, Z-Hunt and Chip-Seq Z-DNA predictions. (**A**) The region chr9:37,800,000–37,805,000—Z-DNA overlaps with the 1st exon of DCAF10 gene; (**B**) the region chr18:67,870,000–67,875,000—Z-DNA is located in the promoter area and overlaps with the first exon of RTTN gene; (**C**) the region chr16:67,905,000–67,910,000—Z-DNA is located in the regulatory area of two bidirectional genes EDC4 and NUTF4; Z-DNA overlaps with promoter area, 5′UTR and the first exon of EDC4 and upstream area of NUTF4 gene; (**D**) The region chr1:565,000–570,000 has a high potential to form Z-DNA; four non-coding RNAs are located in the region.

Examples of DeepZ, Z-Hunt and Chip-Seq Z-DNA predictions for several regions are presented in Fig. 7. Chip-seq predicted regions are presented as green rectangles, DeepZ are predictions marked as blue profiles. In two cases Z-DNA is located in the promoter regions of genes DCAF10 and RTTN, overlapping with 5′ UTR and the first exon of the both genes (Fig. 7A,B).

In Fig. 7C we show de novo DeepZ prediction of Z-DNA. The prediction Z-DNA region is located in the regulation region of two bidirectionally transcribed genes EDC4 and NUTF2. Z-DNA overlaps with promoter area, 5′ UTR and the first exon of EDC4 and upstream area of NUTF4 gene. Z-Hunt prediction is marked with a red line. Experiment did not detect Z-DNA in this region. We also show the case when DeepZ predicts a large area (of 5000 bp) with a high potential to form Z-DNA (Fig. 7D). This region does not contain genes but instead have 4 non-coding RNAs. More examples on DeepZ Z-DNA predictions can be found in Supplementary Fig. S2.

### Feature importance analysis

**Figure 8:**
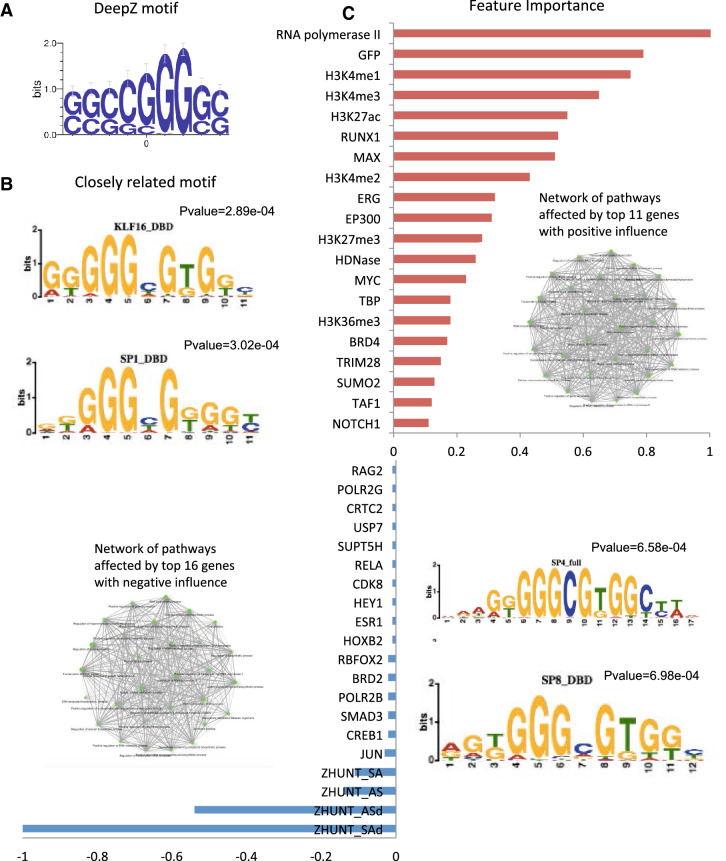
Feature importance analysis. (**A**) DeepZ-predicted significant DNA motif (B) List of DNA-motifs for known TFs showing significant similarity to DeepZ motif (motifs are generated with Tomtom^48^ from The MEME suit^49^) (**C**). Positive and negative normalized regularization coefficients (top-20 positive and top-20 negative are presented, the full list can be found in Supplementary Table S3). Networks are generated with ShinyGO^44^.

We performed feature importance analysis to retrieve factors defining the position of functional Z-DNA regions. We used a regularization method that nullifies unimportant predictors (see “Methods” section). We ranked the features in order of importance normalizing by the maximum value of the highest weight for positive and negative values (Fig. 8C). The full list of features with corresponding regularization coefficients are given in Supplementary Table S4. The important finding is that the energies of B-Z and Z-Z junctions that were used by Z-Hunt were revealed as having the maximum absolute weights. The negative sign reflects the fact that the higher the difference between the energies between B- and Z-DNA, the lower is the probability for a region to adopt Z-DNA conformation. Among all reported features, some factors are known as associated with Z-DNA, such as HIF1A, SLC11A1, ARNT, TRIM28 and SUMO2^9,24,45–47^. As it can be seen from the enriched pathway network depicted in Fig. 8C these top-10 influential genes affect many pathways participating in many cellular processes. The highlighted histone marks are associated with active transcription and transcription regulation.

At the sequence level we were able to retrieve a GC-rich motif (Fig. 8A), which is similar to SP family and KLF family of TFs (Fig. 8B). This motif also has a similarity to quadruplex pattern. The detected motif is not exactly GC- or purine/pyrimidine alternating pattern, and its importance in prediction of Z-DNA regions needs further investigation.

## Discussion and conclusions

We propose a deep learning approach, which uses experimental Z-DNA data for training and learns by aggregating information from sequence, B-Z transition energy, transcriptomics, epigenomics, and chromatin organization levels. After testing different deep learning architectures—CNN and RNN, we chose RNN-based architecture and built the DeepZ model. DeepZ was trained on ChIP-seq data from Shin et al.^9^ and ssDNA data from Kouzine et al.^32,33^ and Wu et al.^33^ that were overlapped with Z-Hunt predictions. The model utilized sequence information together with information on B-Z transition energy and aggregated information from around 30,000 datasets on histone marks, DNase I hypersensitive sites, transcription factor and RNA-polymerase binding sites, and various DNA methylation and DNA methylation variants maps. With DeepZ we performed predictions of Z-DNA regions in the entire genome by assigning probabilities to a region to form functional Z-DNA.

We compared prediction power of Z-Hunt and our model DeepZ on experimental Z-DNA mapping data set: ChIP-Seq^9^ and DeepZ outperforms the computer program Z-Hunt and also predicts many novel potentially functional Z-DNA regions. Z-Hunt algorithm is based on physical and chemical models of B- to Z-DNA transitions taking into account experimental measurements of dinucleotides adopting *syn* and *anti* conformations, and on the assumption that Z-DNA favors alternating purine-pyrimidine patterns. However the experimental data showed that many non purine-pyrimidine alternated sequences can form Z-DNA^50^, and thus other factors influence Z-DNA formation^9^. Z-Hunt model predicts that potentially around 400 000 regions can adopt Z-DNA conformations, and clearly they are not all functional. The functional Z-DNA regions located in promoter or gene areas can serve as transcriptional regulators, both as activators and repressors (examples are cmyc, HO-1, DAI), and they also have characteristic histone and regulatory code. The main idea underlying our approach is to take advantage of the available omics data and retrieve hidden information from epigenetics and regulomics for determining functionality of Z-DNA. The advantage of deep learning model is that the network learns itself important features both from the sequence and from epigenetic and regulatory code.

The Shin data set is enriched in promoters and genes however 24% of all predicted Z-DNA were found in intergenic regions. Indeed, the most studied function of Z-DNA is transcription regulation, however other Z-DNA functions were found, such as nucleosome and chromatin active domain boundaries^6,32,51,52^. Z-DNAs were found to be enriched in centromeres, which in turn are enriched in scaffold/matrix attachment regions. The existing whole-genome Z-DNA sets reflect only a small fraction of all functional Z-DNA in the genome that was detected by Z-DNA-binding proteins (here ADAR modified with two Z-DNA-binding domains). Functional Z-DNA is formed dynamically. Potential Z-DNA regions change conformation from B to Z in response to a signal, and then must change it back to B-form. Dynamic formation of Z-DNA in response to fear was observed, and Z-DNA serves as a fast regulator of gene expression levels^53^. Moreover this process is reversible in a short period of time, and level of Z-DNA in the locus of interest was reduced after fear extinction training. This finding is inline with the general concept of flipons, regulatory genomic elements encoded by non-B DNA structures that are capable for quick dynamic regulation of the transcriptome^54^.

Z-DNA plays an essential role in type I interferon responses^23^. We found that almost half of interferon response genes from the study of interferon type I response^43^ have Z-DNA in promoter regions. GO-enrichment analysis of all DeepZ-predicted genes with Z-DNA in promoters revealed cellular localization and cellular response to stress among significantly enriched gene functional categories.

All the diversity of Z-DNA functions only starts being unfolded. The protein ZBP1 with Z-DNA-binding domain was shown also to function as foreign DNA/RNA sensor, and the sensing is done with Z-DNA-binding domain^55,56^. RNA-editing enzyme ADAR1 contains even two Z-DNA-binding domains, Zα and Zβ, and the their functional role remains largely unknown^57^. Z-DNA binding motif was found in 182 proteins Zα mostly orthologs of ADAR1, ZBP1, E3L, PKZ^18^, and a number of viral proteins that have a functionality in innate immune response^58^.

The neural network that lies at the basis of DeepZ captures not only sequence composition but also another layer of genome organization—epigenetics. Histone code defines positioning of active transcription sites, chromatin partitioning into open/close state and chromatin active domains. ChIP-seq detected Z-DNA regions were strongly correlated with the H3K4me3 and H3K9ac marks, both are marks of active transcription. Our feature importance analysis revealed five important histone marks: H3K4me3, H3K27me3, H3K27ac, ZK4K7K11ac, and H4K5K8ac (with anti sign). The H3K4me3 mark is also associated with open centromeres H3K4me3^59^ where Z-DNA hotspots were found in ChAP-seq experiment, and our DeepZ model confirmed it too. Our model selected enhancer H3K27ac and negatively selected superenhancer mark H4K5K8ac^60^. The other two marks H3K27me3 and H2A.ZK4K7K11ac are mostly associated with gene deregulation by chromatin remodeling, though the last has many controversial functions^61^.

Another epigenetic factor—methylation—also is interrelated with Z-DNA formation. It can facilitate B- to Z- transitions specifically for dinucleotide d(GC)(5) repeat sequence^62^. However, our approach did not reveal methylation and its variants as highly significant contributors to the model prediction power but this can be explained by the lack of experimental data on methylation variation maps.

Important features from regulatory code are also consistent with earlier findings. For example HIF1A binds with Z-DNA region in the promoter of SLC11A1 gene, which expression is associated with susceptibility to infectious diseases^45,46^. Others Z-DNA associated proteins were reported: ARNT45^9^, TRIM28^24^, and SUMO2^47^.

Information from sequence level retrieved GC-rich motif (Fig. 8). The resulting sequence is very similar to the purine-pyrimidine pattern d(GGGC), that have been shown to adopt a Z-DNA conformation^50,63^. However this motif should not be regarded as the only sequence motif associated with Z-DNA formation. This is the motif that statistically more often appeared in the analyzed data set. First, it is retrieved from the neural network trained on the small ChIP-seq data set of Shin et al.^9^, and the data set has its biases. Secondly, the length of the obtained motif is restricted by the network architecture, i.e. by the vision range of CNN filters. When more data on Z-DNA become available, more DNA motifs associated with Z-DNA formation will be discovered.

An assessment of the role of Z-forming Alu and GT repeats is not fully assessed in the results presented. This is due to a limitation from the exclusion of Alu sequences in the initial processing of sequence datasets and due to the absence of GT-rich sequences in the experimentally available Zα Chip-seq datasets. The DeepZ approach can be applied to these datasets as they become available to make additional predictions. It will help further to reveal the role of Zα and the Z-duplex in editing of Alu repeat elements and will further the understanding of HIF1α, which is reported to bind to GT sequences as well as other Z-motifs similar to those identified here^45,64^.

An advantage of our approach is the combination of sequence-intrinsic information with higher level mark-ups (epigenomics, transcriptiomics, chromatin organization). Our attempts to predict Z-DNA regions with deep learning models based on sequence information only were not successful (the results are not shown here). We predict regions that are similar in structure and functional arrangement of epigenetic and functional genomic factors that were not detected in the ChiP-seq 2016 experiments just because these genomic regions were closed at this particular moment in this particular cell line. However these regions could become functional at some other conditions. The proposed approach is not restricted to the data sets available as of today and is applicable when more experimental omics data become available. We assign probabilities to Z-DNA regions that will be refined when more information is taken into account.

In summary, DeepZ is the first deep learning model applied to the task of predicting Z-DNA regions. The fact that a deep learning model, benefitting from epigenetic and regulatory code, can efficiently recognize Z-DNA regions points to the regulatory potential of Z-DNA that is yet to be fully discovered. The proposed whole-genome annotation with potential Z-DNA regions will be useful to many researchers studying the role of Z-DNA in genome functioning.

## Methods

### Input data

There were two Z-DNA data sets used in this study. The first data set is composed from CHiP-seq experiment^9^ that reported 391 Z-DNA regions for the training set for machine learning models. The second data set is composed of data from Wu et al.^33^ and Kouzine et al.^32^. All data sets were cleaned from ENCODE blacklist regions^41^. Z-DNA regions were encoded to a boolean array, where 1 is assigned to nucleotides in Z-DNA regions and 0 otherwise.

Widely used approach in bioinformatics is to consider the task as a classification problem, where the class is predicted based on interval features. With this approach our task is unsolvable due to the lack of the minor class elements. We consider the task as a segmentation problem instead. Thereby every Z-DNA region becomes a set of the minor class elements with an average size of 400 nucleotides. Thus the size of the minor class exceeds 150,000 elements, what is already enough for the usage of deep learning methods. In the result section we demonstrated that this approach solves a generalization problem. However, the ratio between the number of nucleotides containing a Z-DNA site and the number of nucleotides out of Z-DNA region is approximately 1 to 50; that is why all target metrics are resistant to class imbalance.

Apart from the initial sequence information we integrated in the model B-Z transition energy from Z-Hunt (see Table 2 in^34^) and the additional information on histone marks (HM), DNase I hypersensitive sites (DNase-Seq), transcription factor (TF) and RNA-polymerase (RNAP) binding sites. Methylation variation maps were taken from^65^. First, the primary DNA sequence was encoded using one hot encoding (OHE) technique. Each chromosome is mapped to a matrix of size L ✕ 4, where L is the length of a chromosome. Then information about epigenetic and regulatory code was added as described below. All available CHiP-Seq data were downloaded from Chip Atlas^66^ with the lowest threshold for significance equals 50. The information on one type of marker from different tissues was aggregated as the presence of the marker in any tissue. The total set included 1058 markers of which 100 histone marks, 947 transcription factor binding sites, 10 RNA-polymerase binding sites and DNase I hypersensitive sites. Totally 1058 features were selected. The full list of features is given in Supplementary Table S1.

Each feature is linearly scaled to the interval [0, 1] and has the length equal to the length of a chromosome. Thus, each chromosome can be mapped to the matrix of size L ✕ 1062. All matrices for all chromosomes were concatenated, so that the matrix data can be perceived as images of dimension 1, where 1062 features correspond to different color channels.

The total size of the human genome exceeds 3 ✕ 10^9^ nucleotides. If every value of the matrix is encoded by 4 bytes float, the total volume of memory consumption will take 3 terabytes of RAM. This volume is unrealistic for modern computers. To overcome this problem, the data can be either stored on a hard disk or the data can be extremely compressed and loaded into RAM memory. In this work we use the second approach. To compress this data, we implemented a special container. After compression, the data took up only about 200 megabytes. Hereby all the input data can be run in RAM memory permanently. The implementation is available in the repository https://github.com/Nazar1997/Sparse-vector (see also Supplementary Methods for details).

Z-DNA regions were encoded into the boolean array, where 1 is assigned to nucleotides in Z-DNA regions and 0 otherwise. Likewise the input matrix, the target vector has the length of a chromosome.

### Train and test set

Every chromosome sequence was divided into a set of subsequences. In this work we avoided generation of boundaries of subsequences based on the sites of Z-DNA, so every chromosome was evenly cut into pieces with the length of 5000 nucleotides. For train and test sets we included all subsequences containing Z-DNA and background sequences that do not contain Z-DNA, which were randomly chosen from the entire genome. Randomization was fixed for reproducibility. The number of non-Z-DNA sequences was triple the number of Z-DNA-containing sequences. Training and test sets were stratified and divided in the ratio of 4 to 1. The stratification was based on Z-DNA presence and chromosome number.

### Deep learning architectures

In this paper we tested 151 different architectures comprising three types of deep learning approaches: CNN, RNN, and hybrid CNN-RNN. Every deep learning model consisted of main building blocks: convolutional (CNN) and/or recurrent (RNN), and fully connected (FC) blocks. Every block could be represented by more than one layer. Every model has at least one FC layer. We tried a different number of FC layers with a dropout layer in between. Probability of every dropout layer was set to 0.5. The last FC layer has two output neurons, first corresponding to 0 output, and second to 1 (see also Supplementary Methods for details).

#### Deep learning architectures based on CNN

This type of DL models consists of only CNN and FC (Fully Connected) layer blocks (Fig. 1A). One and two CNN layers with ReLU activation in between CNN layers were tried. Number of convolutional kernels and kernel size varied from 1 to 17. Stride was set to 1, padding was set to (kernel size 1)/2, to keep the same size of the output. Every convolutional kernel has 1D conformation. An output of the CNN block is sent to the FC block, where final prediction is made. In total 54 CNN-based architectures were tested.

#### Deep learning architectures based on RNN

This type of DL models consists of only RNN and FC blocks (Fig. 1B). Untouched input is sent to the RNN block. The RNN block consists of the LSTM network with different hyperparameters. We tested one and two LSTM layers, one and bi-directional LSTM with various hidden sizes. Output of the RNN block is sent to the FC block where final prediction is made. Totally, 65 RNN-based architectures were tested.

#### Hybrid deep learning architectures based on CNN and RNN

This type of DL models consists of both RNN and CNN, and FC blocks. The input is first sent to the CNN block, then to the RNN block, and the final prediction is made in the FC block. Searching for hyperparameters for each block was the same as described above. In total 32 hybrid CNN-RNN architectures were tested.

### Training parameters

All models were trained using RMSprop via backpropagation. RMSprop is the unpublished, adaptive learning rate method proposed by Geoff Hinton. Instead of the full-gradient calculation, the gradient was calculated on a subset of the training set. After each gradient calculation model parameters were updated accordingly (see also Supplementary Methods for details).

### Whole-genome annotation with Z-DNA regions

The entire data set was divided into fivefolds of equal size and each fold was stratified by chromosome number and Z-DNA presence/absence. At each step, onefold out of 5 was set as a test set and the DeepZ model was trained on the remaining 4 folds. In total, 5 DeepZ models were trained and each model performed predictions for the remaining genomic regions. The final probabilities for a nucleotide to belong to a Z-DNA region was calculated as an average of all five model predictions. Thus every nucleotide from every chromosome was assigned a probability to belong to a Z-DNA forming region. The cutoff threshold (0.343) was chosen as a value that maximizes F1 score on the united set of all 5 folds. Nucleotides with the probabilities higher than the threshold were combined in regions. All intervals with a gap less than 11 bp were joined together and all intervals shorter than 11 bp were skipped, taking into account that 11 bp is the length of one turn of DNA helix.

### DeepZ model interpretation

Here we aimed to determine features that the model considered as important and secondly to extract information about DNA sequences that the model considered as important for the prediction of the Z-DNA sites. In order to find the important features, the model with high regularization penalty has to be trained. Since RNN architectures are not good for interpretations, we used the best CNN model, which performance was only slightly inferior to the best RNN model. The selected model has a quality of more than 80% ROC AUC and consists of 2 convolutional layers, each having the kernel size of 5.

**Figure 9:**
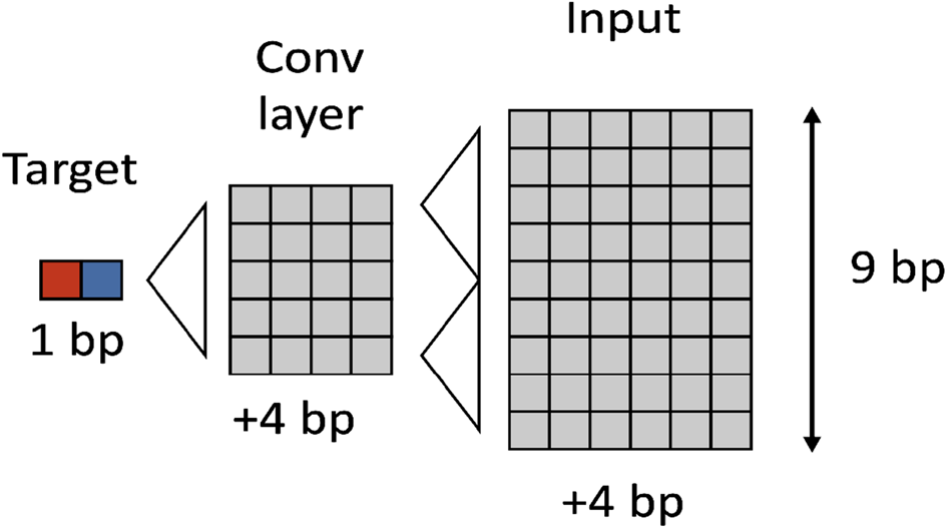
Model interpretation scheme.

In the previous research for image classification task, the authors computed the gradient of the class score with respect to the input image^67^. In our work, the method is similar but the input is a 1-d image of the DNA sequences, the target is Z-DNA. The training of the CNN model was similar to the RNN model with an addition of 10^−3^ and 10^−2^ weights of L1 regularization in the loss function. L1 regularization has the property of nullifying all unnecessary model weights. All the features that have zero weights in the first convolutional layer are further ignored, the weights of the model trained this way are frozen, and then the trainable input is passed again to this model. The structure of the model allows limiting the trainable input length to 9 nucleotides (Fig. 9). The most distant filter of the 2nd layer is located at a distance of 2 nucleotides, in turn the most distant nucleotide is located at a distance of 2 nucleotides from the side filter. Thus, the dependence on the target nucleotide will not exceed 4 nucleotides to the left and to the right. A sequence of 9 elements will completely define one output of the trained CNN model as shown in Fig. 8.

However, unlike a neural network, whose weights can take any real value, values of this input can only take values from 0 to 1. In order to find features that from the model's point of view increases the probability of Z-DNA presence, we have extended the value range from − 1 to 1. This way we can find both positive and negative influencing features. The target function maximizes the predicted probability of becoming a Z-DNA site for the central nucleotide. The same RMSprop with learning rate 10^−2^ was used for input learning. After every learning iteration input values are clipped to the interval from − 1 to 1.

After the input that maximizes the output of the CNN is found, it is difficult to find a DNA sequence that corresponds to its maximum output, since the sequence itself is encoded by the OHE method. This means that all 4 input features depend on each other and their independent maximization can give an incorrect answer unlike other features. In order to find such a sequence, a separate maximization was performed for the encoded sequence, but with additional restrictions. The sum of 4 features for each nucleotide is equal to 1. With these restrictions, the problem is not solved by an ordinary gradient descent, but solved using sequential least squares programming^68^. The output is the weight matrix, which is interpretable as a Z-DNA probability. This may tell us the sequence pattern of Z-DNA.

### Gene ontology analysis

Gene Ontology analysis^69,70^ was done using ShinyGO tool^44^.

## Supplementary information

Supplementary Figure S1.

Supplementary Figure S2.

Supplementary Figure S3.

Supplementary File 1.

Supplementary File 2.

Supplementary File 3.

Supplementary File 4.

Supplementary File 5.

Supplementary Methods.

Supplementary Table S1.

Supplementary Table S2.

Supplementary Table S3.

Supplementary Table S4.

## Data Availability

Codes and results are available at https://github.com/Nazar1997/DeepZ.
