# Supplementary material for: Deep learning approach for predicting functional Z-DNA regions using omics data
Source: Sci Rep. 2020 Nov 5;10:19134. doi: 10.1038/s41598-020-76203-1 (PMC7644757; doi:10.1038/s41598-020-76203-1)

Deep learning approach for predicting functional Z-DNA regions using omics data

Nazar Beknazarov, Seungmin Jin and Maria Poptsova

Supplementary Figure S1. Distribution of DeepZ predicted Z-DNA regions over genomic regions. A. DeepZ trained on Shin data set. B. DeepZ trained on Kouzin-Wu dataset.

A DeepZ Shin

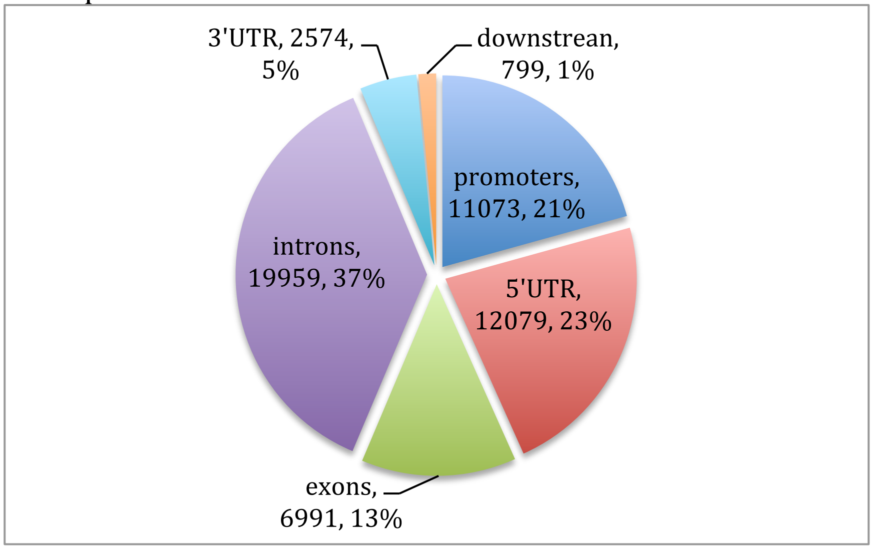

B DeepZ Kouzin-Wu

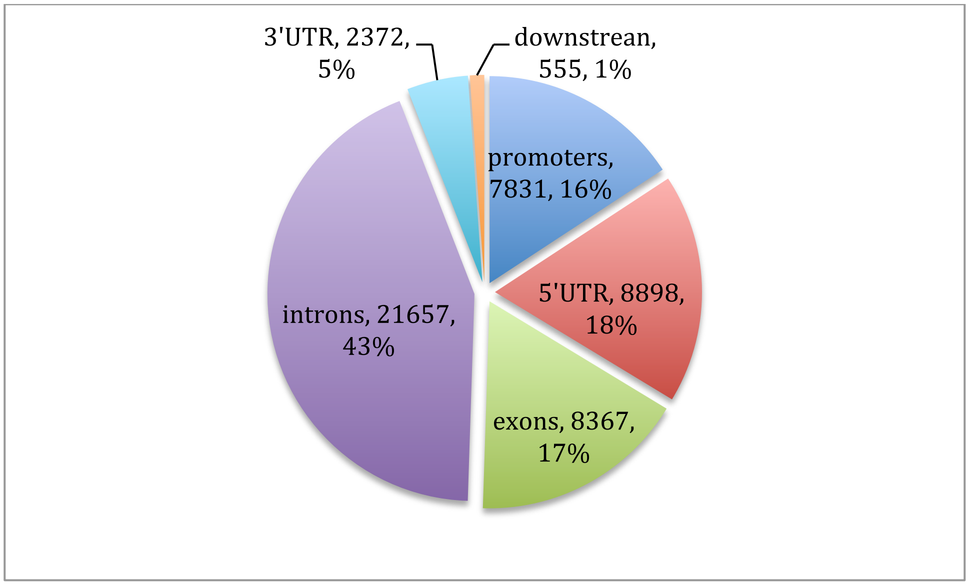

Supplement: Supplementary file 1 — Supplementary Figure S1. [file 41598_2020_76203_MOESM1_ESM.pdf]
