# Supplementary material for: Deep learning approach for predicting functional Z-DNA regions using omics data
Source: Sci Rep. 2020 Nov 5;10:19134. doi: 10.1038/s41598-020-76203-1 (PMC7644757; doi:10.1038/s41598-020-76203-1)
Supplement: Supplementary file 2 — Supplementary Figure S2. [file 41598_2020_76203_MOESM2_ESM.pdf]

Deep learning approach for predicting functional Z-DNA regions using omics data

Nazar Beknazarov, Seungmin Jin and Maria Poptsova

Supplementary Figure S2. GO enrichment analysis of genes with predicted Z-DNA regions in gene bodies (see Supplementary Table S2 for a list of genes in each category); (A) network representation (B) tree representation.

A

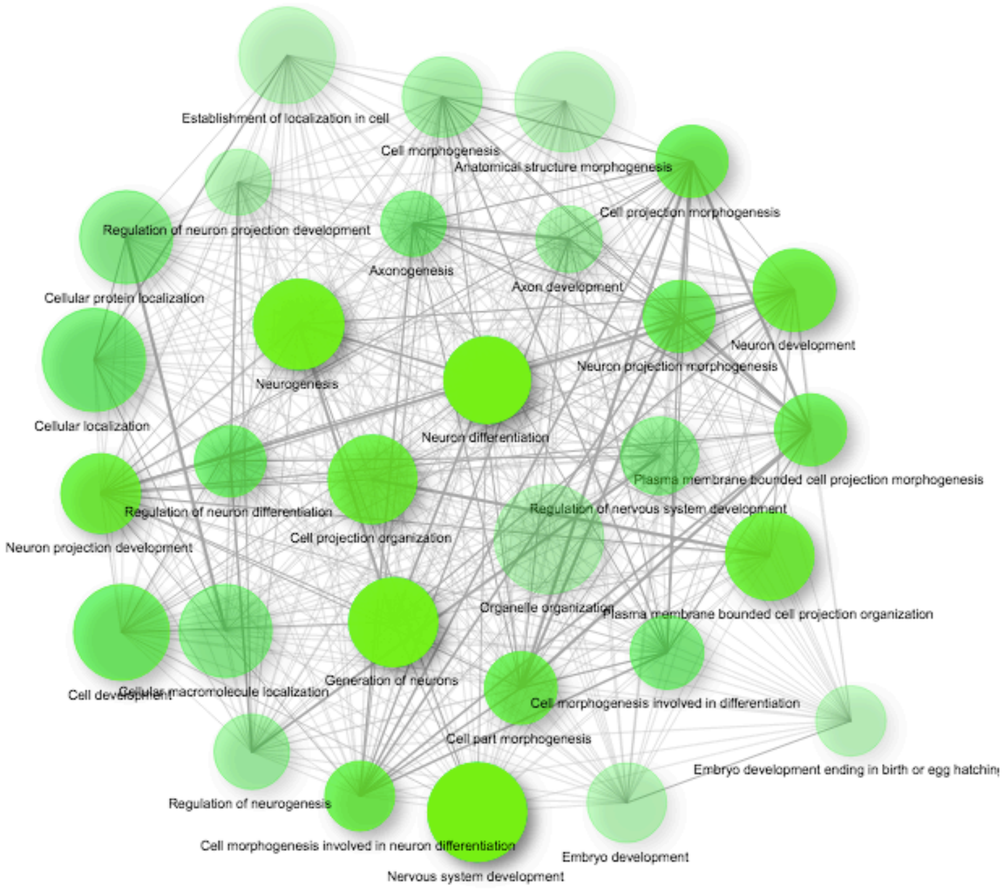

B

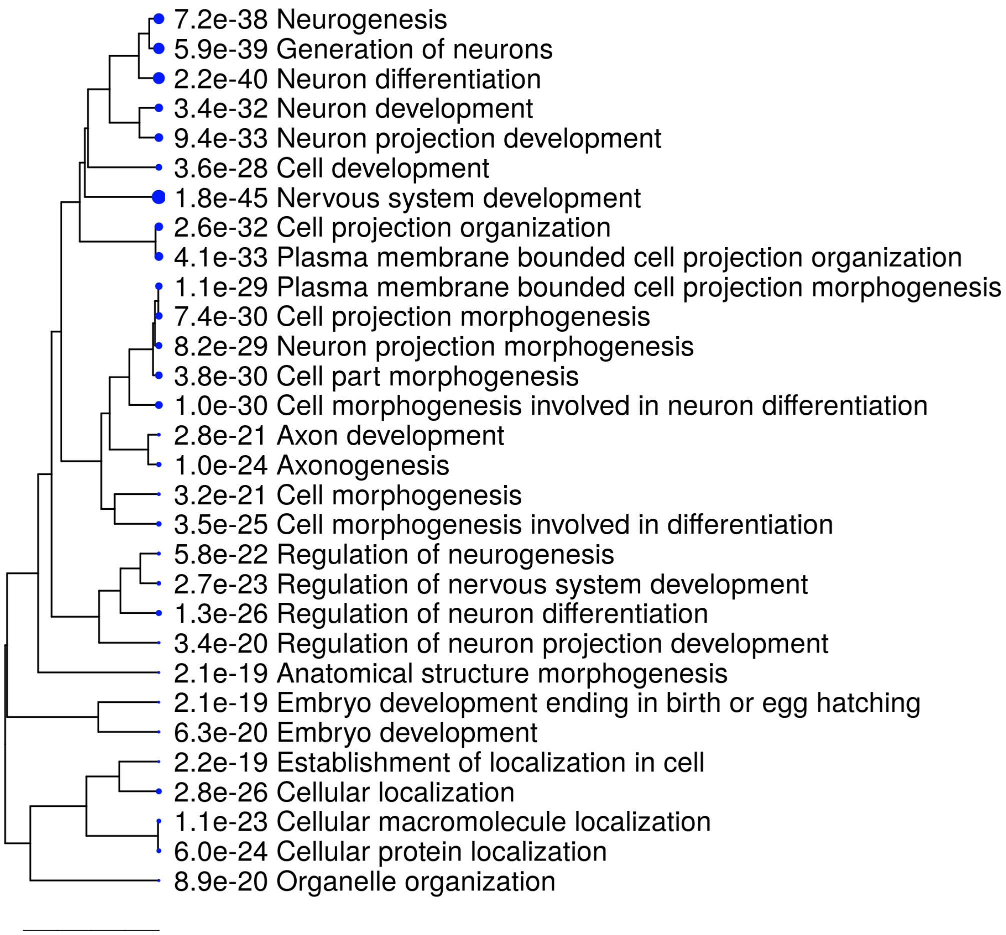
7.2e-38 Neurogenesis
5.9e-39 Generation of neurons
2.2e-40 Neuron differentiation
3.4e-32 Neuron development
9.4e-33 Neuron projection development
3.6e-28 Cell development
1.8e-45 Nervous system development
2.6e-32 Cell projection organization
4.1e-33 Plasma membrane bounded cell projection organization
1.1e-29 Plasma membrane bounded cell projection morphogenesis
7.4e-30 Cell projection morphogenesis
8.2e-29 Neuron projection morphogenesis
3.8e-30 Cell part morphogenesis
1.0e-30 Cell morphogenesis involved in neuron differentiation
2.8e-21 Axon development
1.0e-24 Axonogenesis
3.2e-21 Cell morphogenesis
3.5e-25 Cell morphogenesis involved in differentiation
5.8e-22 Regulation of neurogenesis
2.7e-23 Regulation of nervous system development
1.3e-26 Regulation of neuron differentiation
3.4e-20 Regulation of neuron projection development
2.1e-19 Anatomical structure morphogenesis
2.1e-19 Embryo development ending in birth or egg hatching
6.3e-20 Embryo development
2.2e-19 Establishment of localization in cell
2.8e-26 Cellular localization
1.1e-23 Cellular macromolecule localization
6.0e-24 Cellular protein localization
8.9e-20 Organelle organization
