# Supplementary material for: Deep learning approach for predicting functional Z-DNA regions using omics data
Source: Sci Rep. 2020 Nov 5;10:19134. doi: 10.1038/s41598-020-76203-1 (PMC7644757; doi:10.1038/s41598-020-76203-1)
Supplement: Supplementary file 3 — Supplementary Figure S3. [file 41598_2020_76203_MOESM3_ESM.pdf]

Deep learning approach for predicting functional Z-DNA regions using omics data

Nazar Beknazarov, Seungmin Jin and Maria Poptsova

Supplementary Figure S3. Examples of DeepZ, ZHUNT and Chip-Seq Z-DNA predictions. More examples can be found at https://github.com/Nazar1997/DeepZ.

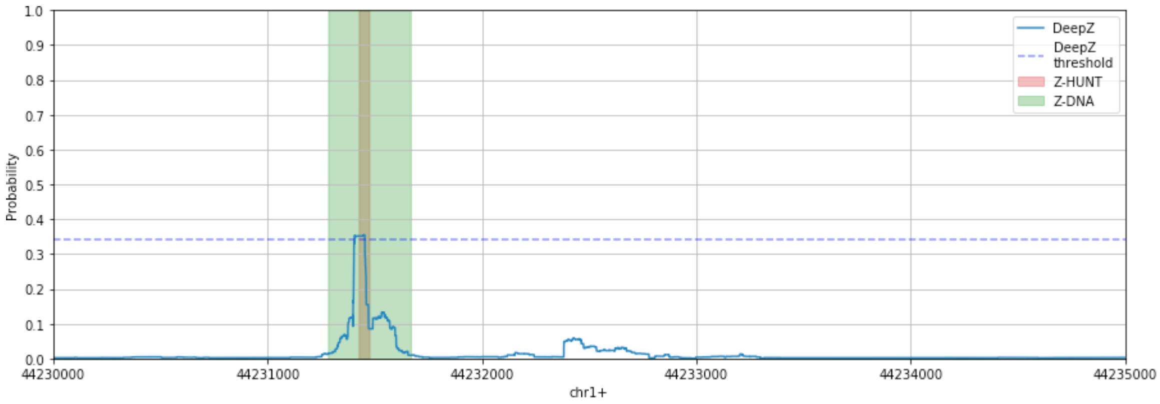

chr1_44230000-44235000

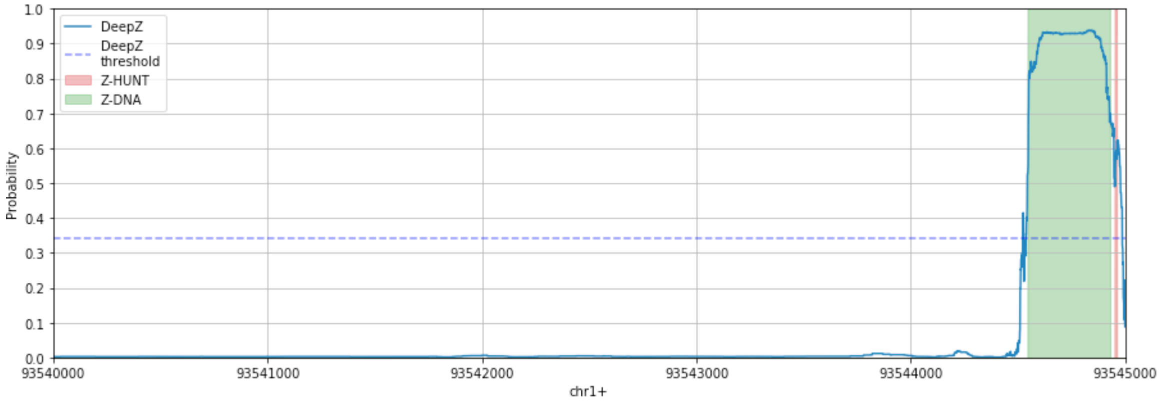

chr1_93540000-93545000

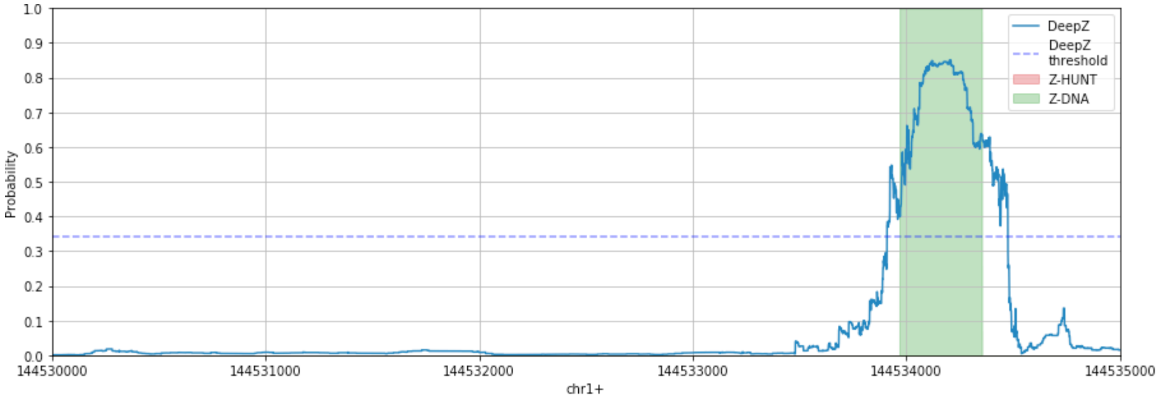

chr1_144530000-144535000

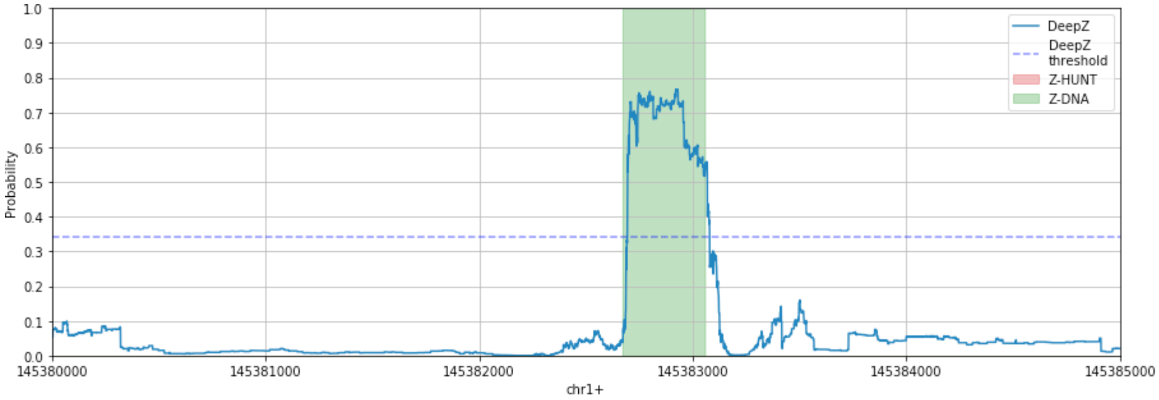

chr1_145380000-145385000

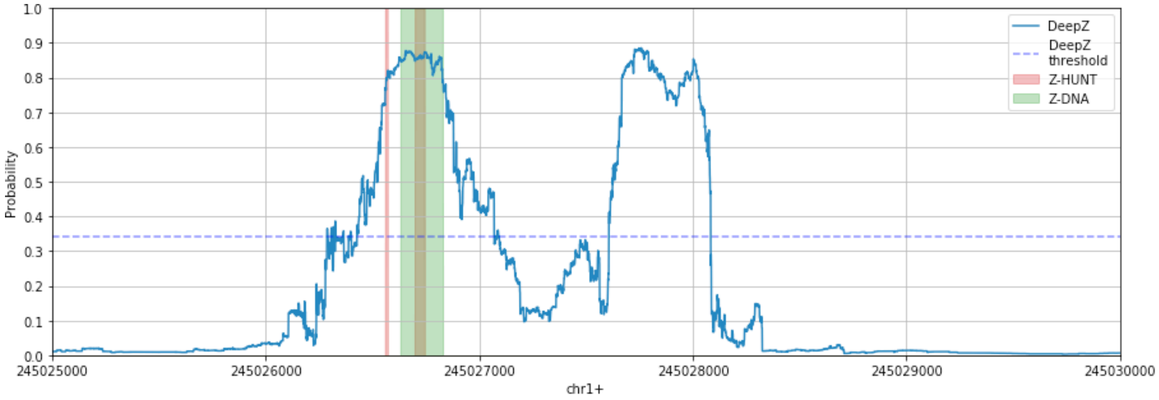

chr1_245025000-245030000

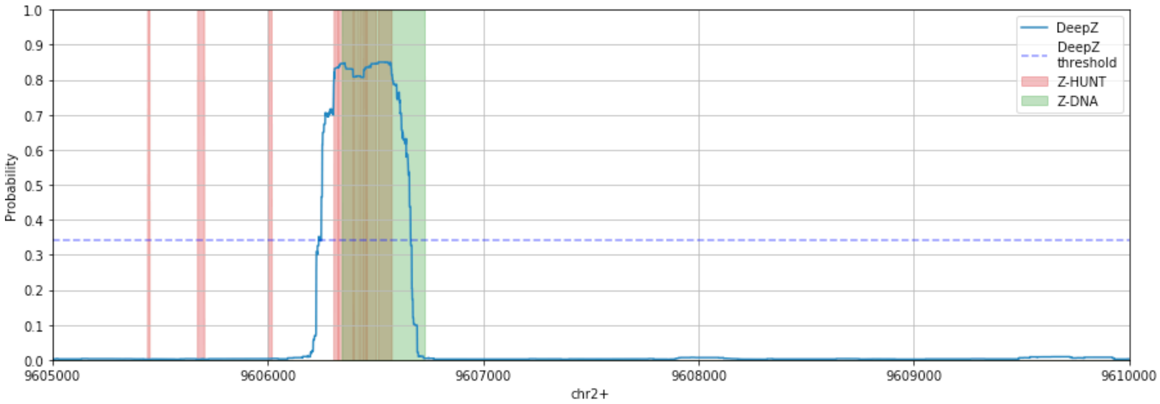

chr2_9605000-9610000

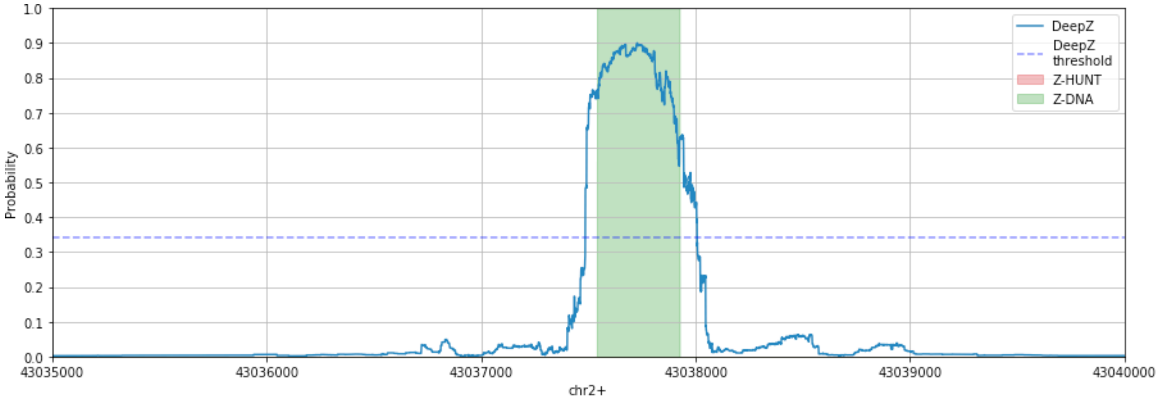

chr2_43035000-43040000

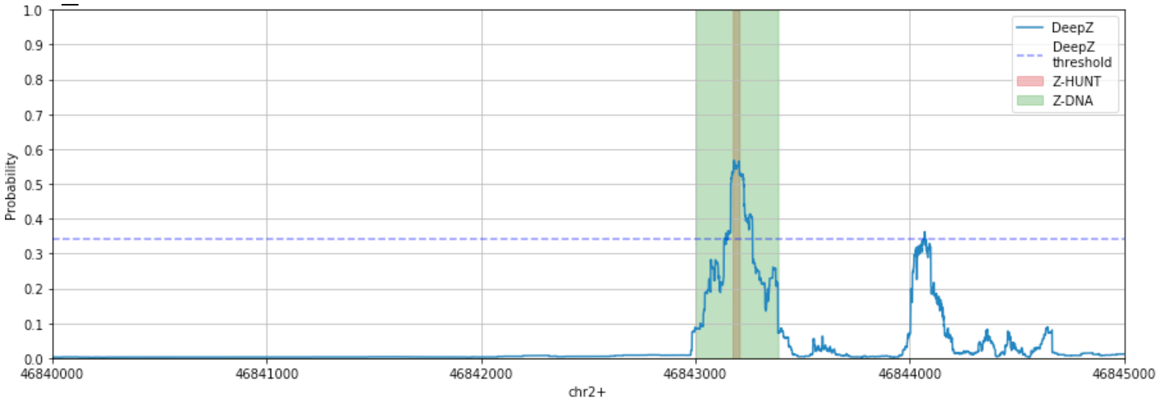

chr2_46840000-46845000

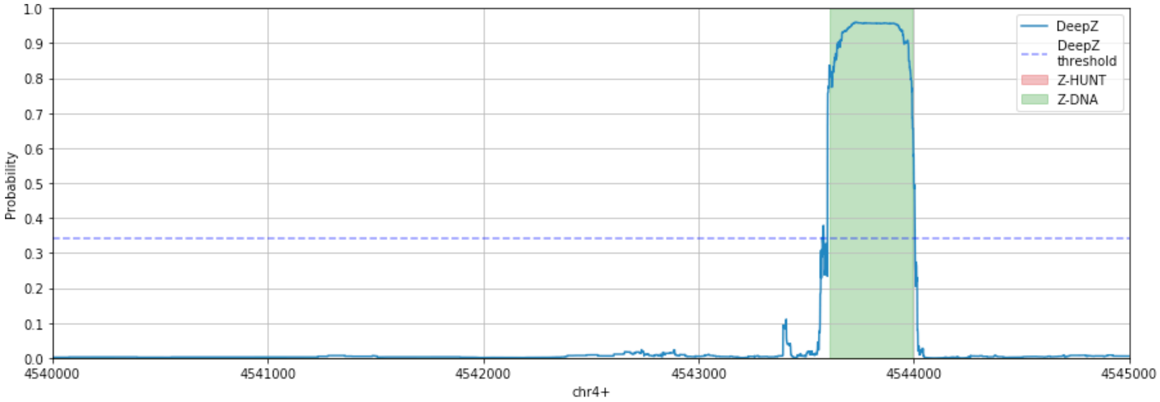

chr4_4540000-4545000

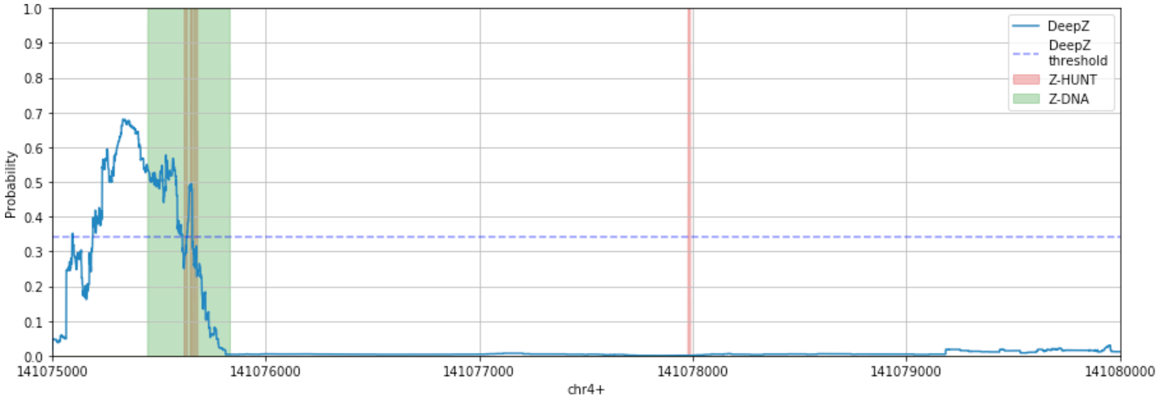

chr4_141075000-141080000

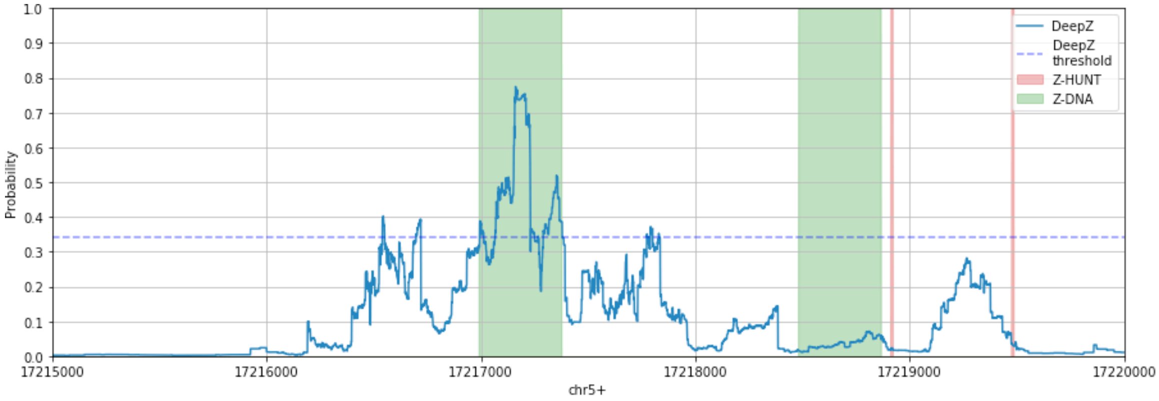

chr5_17215000-17220000

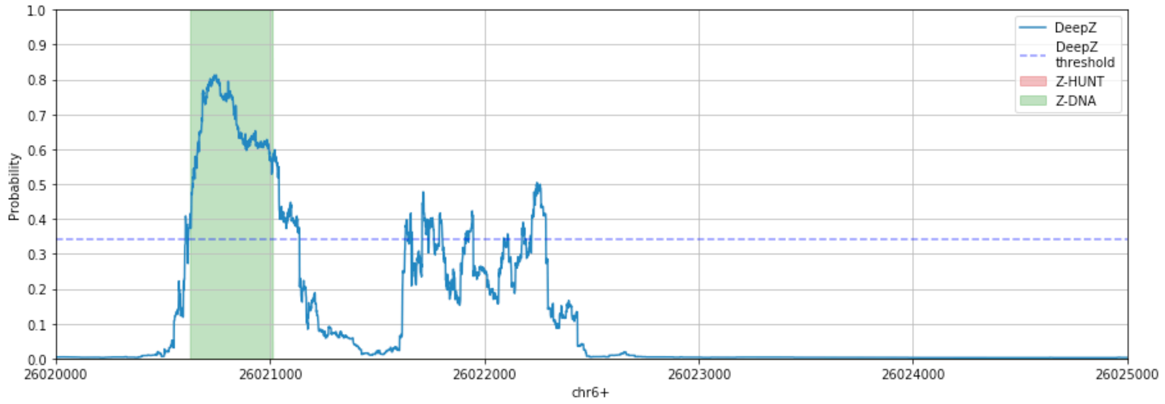

chr6_26020000-26025000

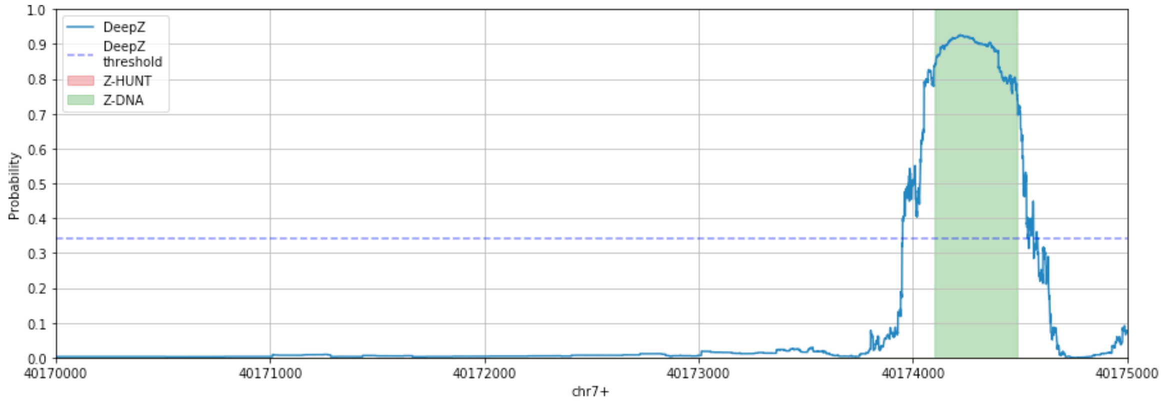

chr7_40170000-40175000

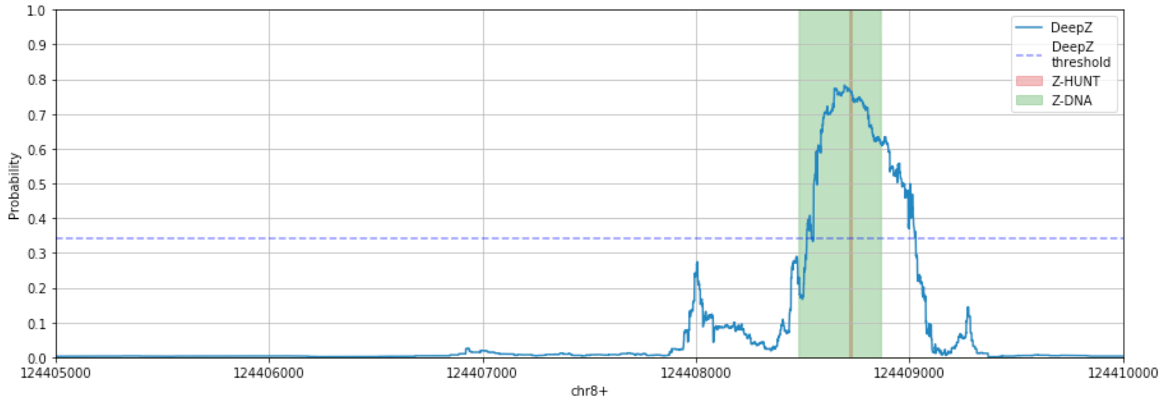

chr8_124405000-124410000

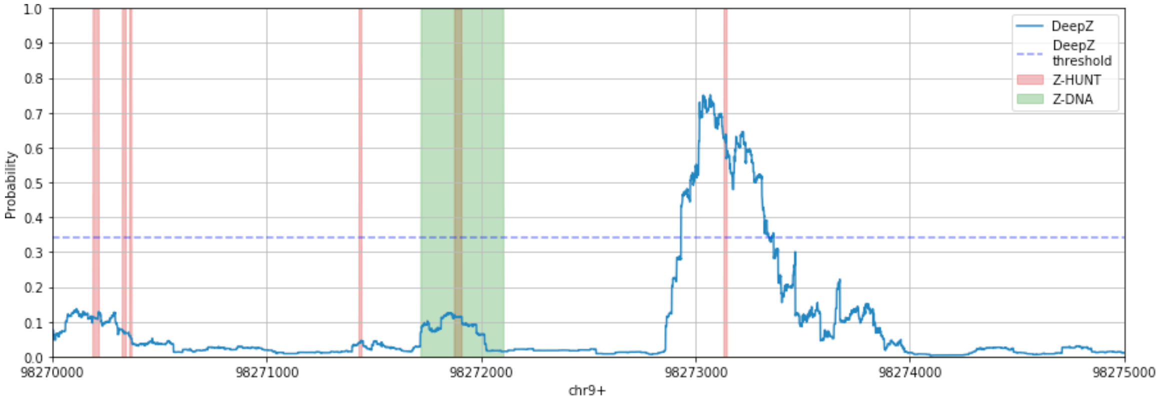

chr9_98270000-98275000

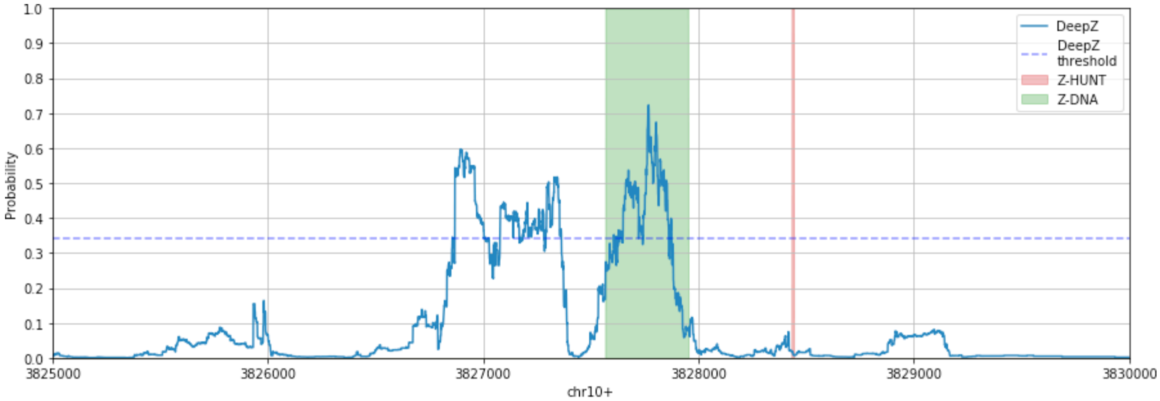

chr10_3825000-3830000

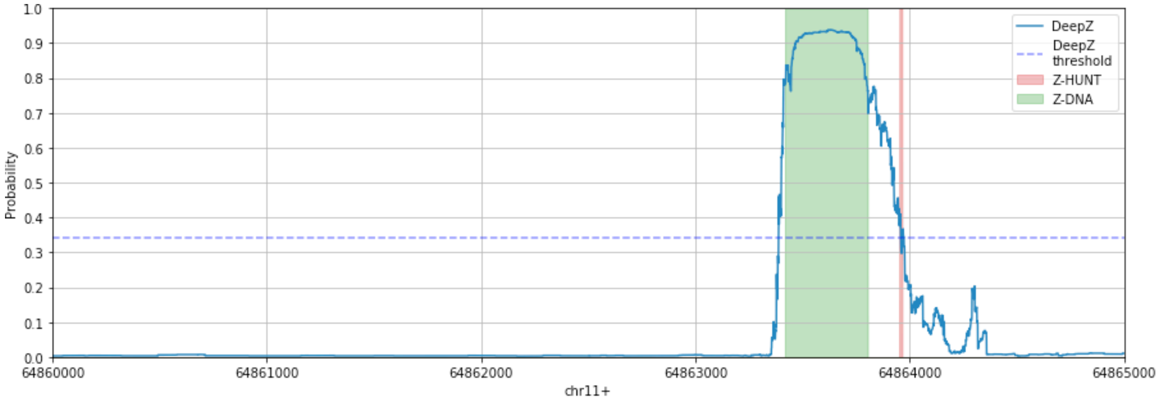

chr11_64860000-64865000

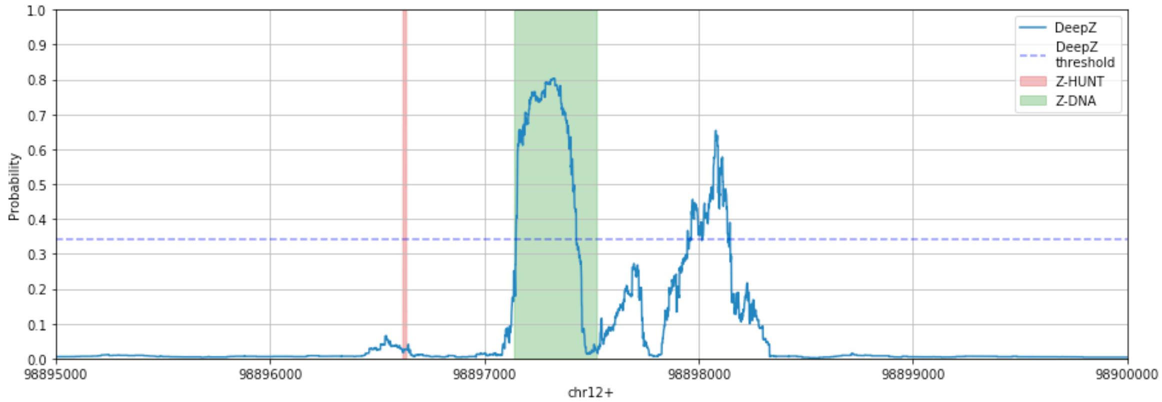

chr12_98895000-98900000

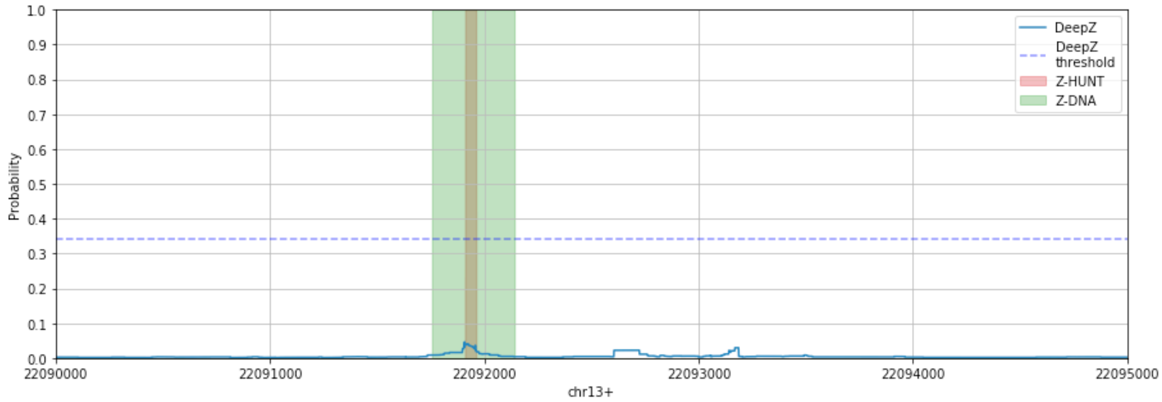

chr13_22090000-22095000

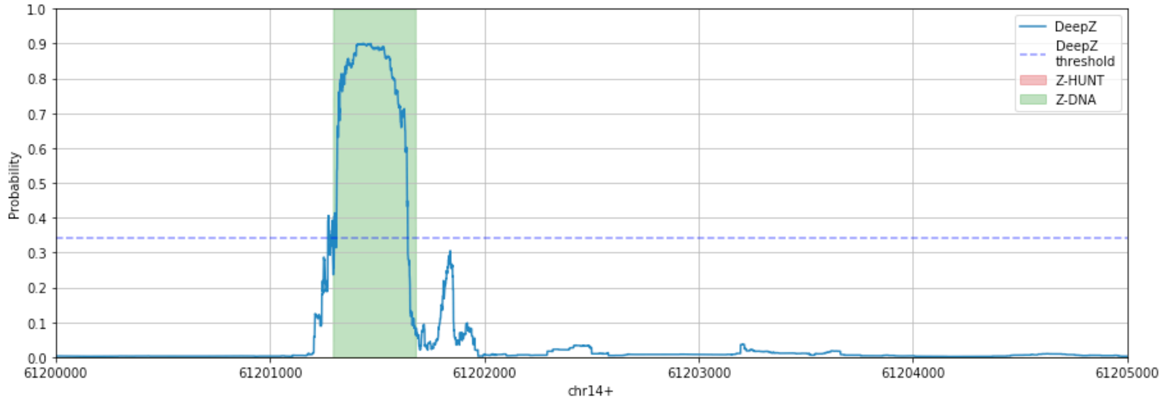

chr14_61200000-61205000

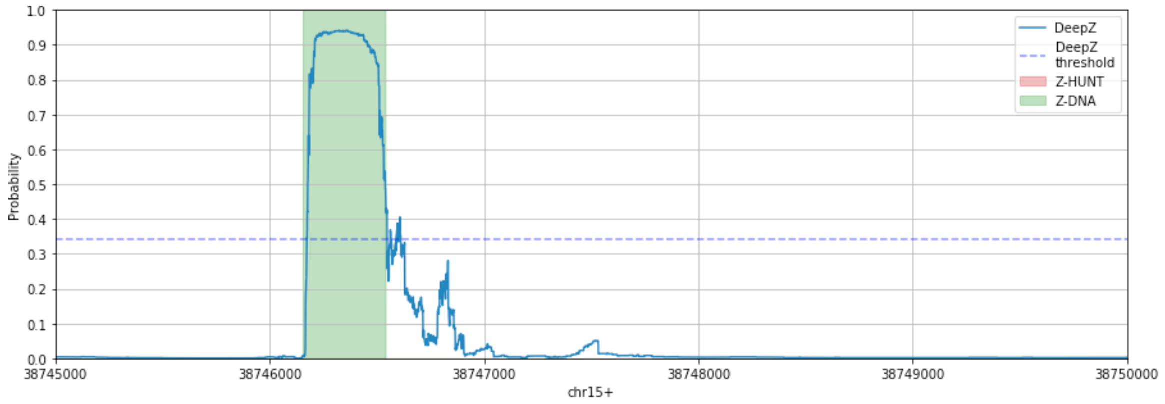

chr15_38745000-38750000

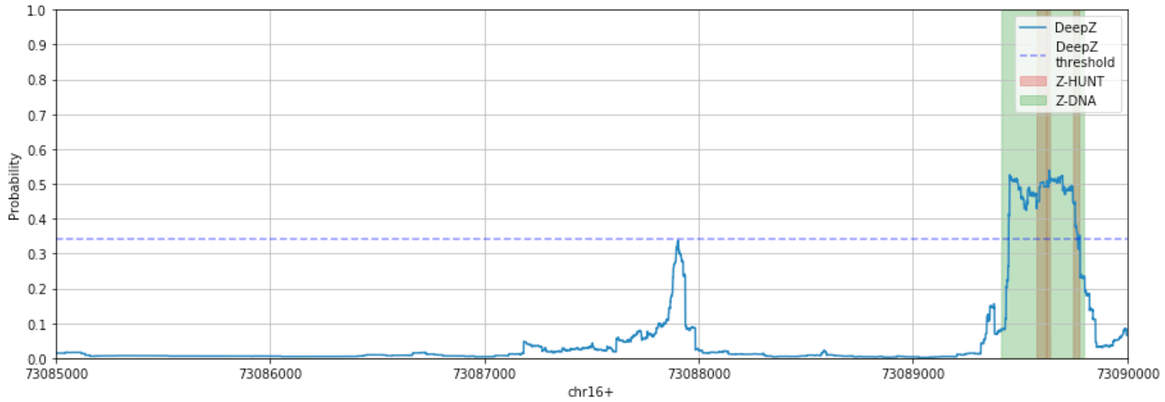

chr16_73085000-73090000

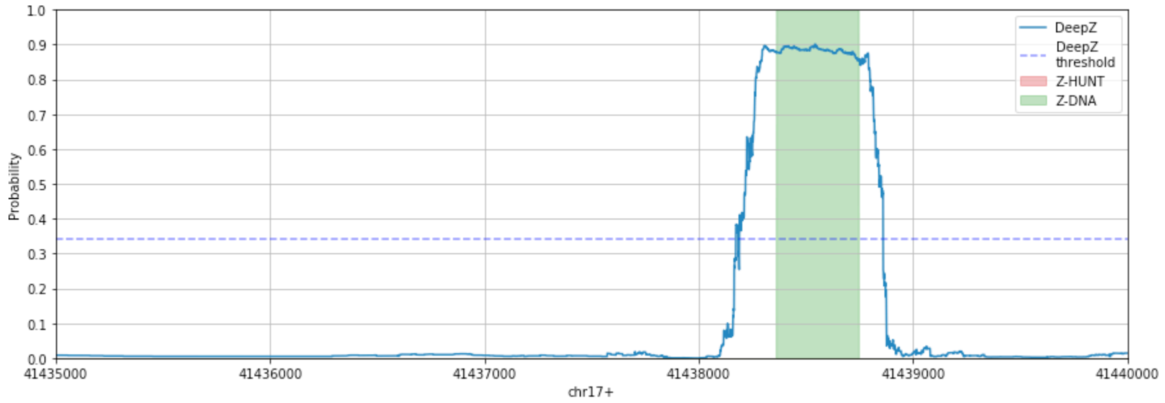

chr17_41435000-41440000

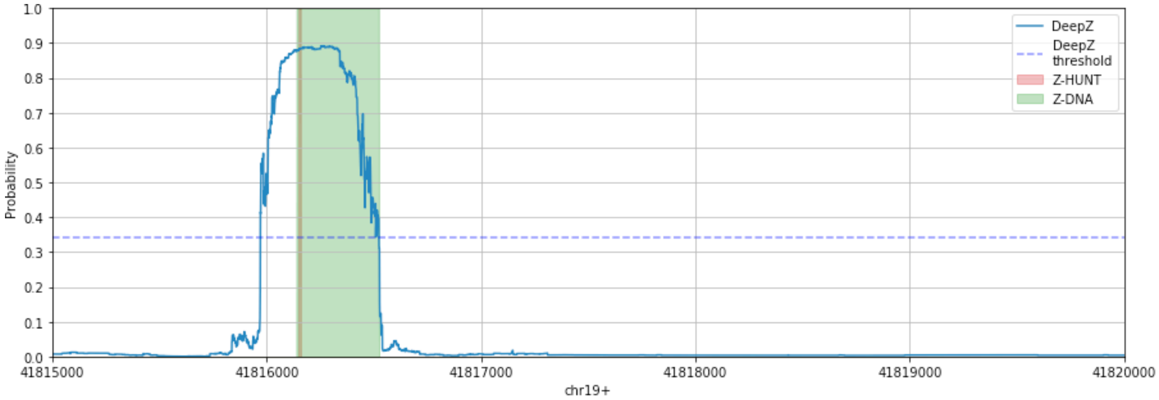

chr19_41815000-41820000

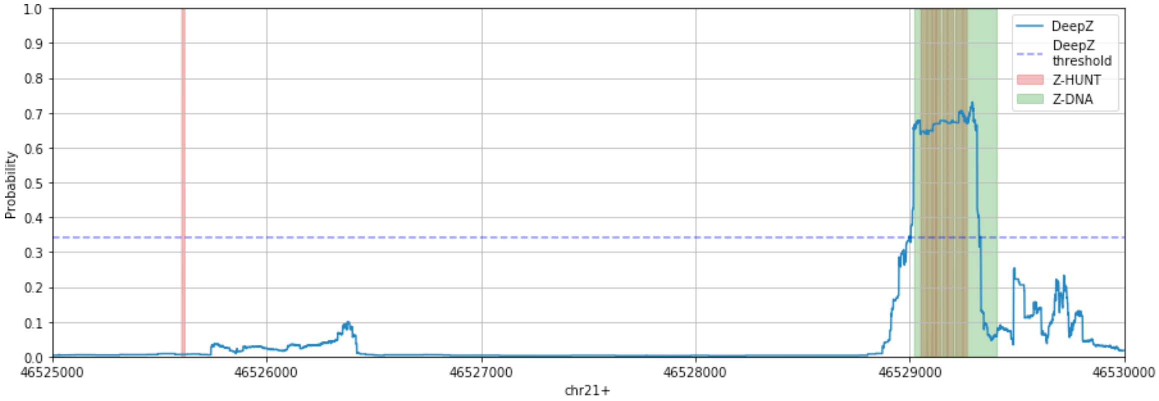

chr21_46525000-46530000

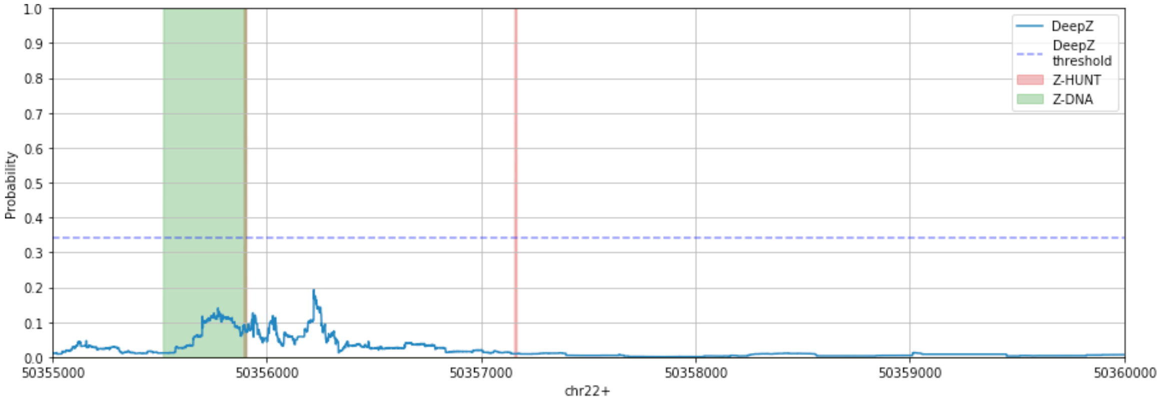

chr22_50355000-50360000

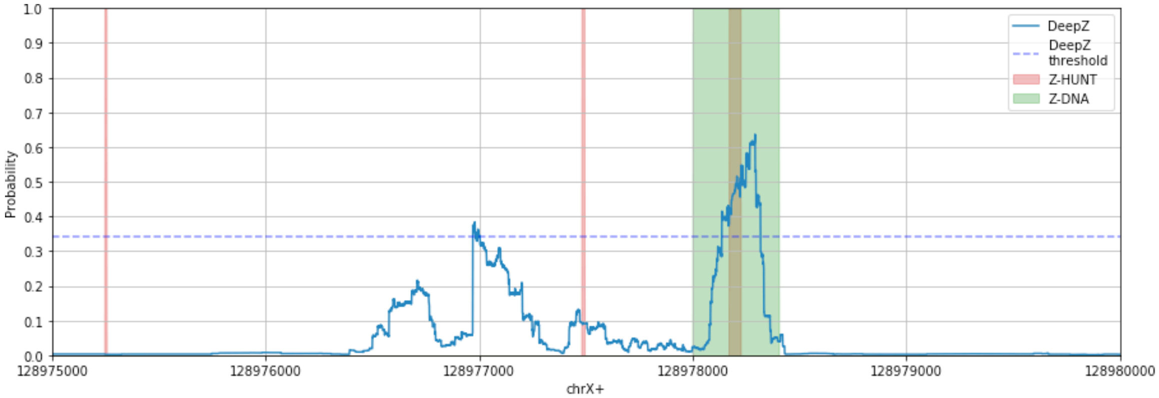

chrX_128975000-128980000

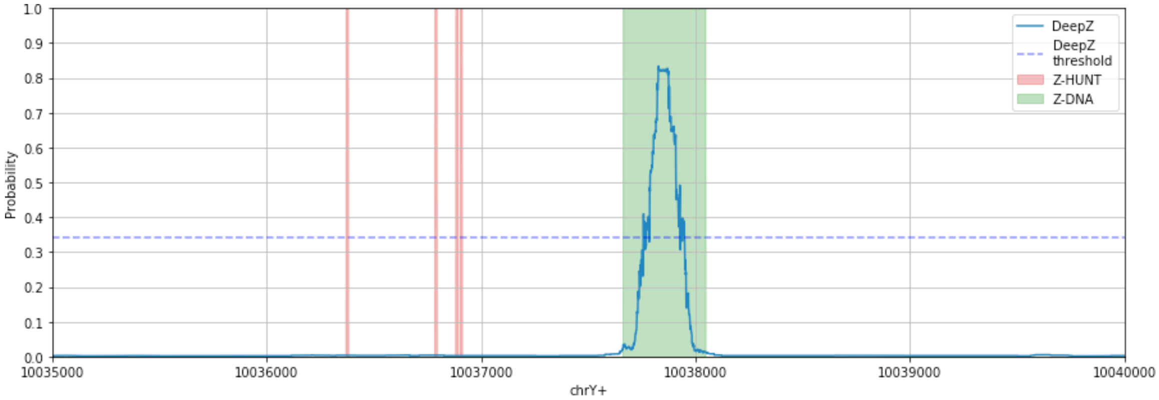

chrY_10035000-10040000
