# Supplementary material for: Deep learning approach for predicting functional Z-DNA regions using omics data
Source: Sci Rep. 2020 Nov 5;10:19134. doi: 10.1038/s41598-020-76203-1 (PMC7644757; doi:10.1038/s41598-020-76203-1)
Supplement: Supplementary file 9 — Supplementary Methods. [file 41598_2020_76203_MOESM9_ESM.doc]

**Deep learning approach for predicting functional Z-DNA regions using omics data**

**Nazar Beknazarov, Seungmin Jin and Maria Poptsova**

**Supplementary Methods**

**Sparse vector realization**

This package was implemented in Python3 using the numpy library. The implementation is available in the open repository <https://github.com/Nazar1997/Sparse-vector>. The basis of this implementation is two vectors. The first data vector stores directly the values of the encoded vector, the second vector stores the indexes of the values in the encoded vector.

This vector supports the following operations:

* Return standard vector values for a given slice [i, j]

* Changing the vector values on a given slice[i, j]

On real data – epigenetic labels, the compression level exceeded 104. Thus, instead of 1 terabytes, one now needs only about 102 megabytes, which is an acceptable level at the moment.

**Training parameters**

All models hyper parameters are specified. All models parameters are initialized by default. Batch size equals to 20. Learning step was set to 10^-4. Weight decay was set to 10^-4. Every model was trained during 20 epochs. All models were trained on 2 GPUs in a distributed manner.

All models were implemented in Python3.6 using package PyTorch1.0. Models were trained on computer with following parameters:

- CPU: Intel Core i7-6950X CPU 3.00GHz 20 Cores
- GPUs: Two GeForce GTX 1080 Ti with SLI between,11 Gigabyte video memory on each of them
- RAM: 62.8 Gigabyte

**Architectures of the best DeepZ models**

The best RNN-based architecture:

- LSTM(1062, 500, num_layers=2, bidirectional=True)
- Dropout(p=0.5)
- Linear(in_features=1000, out_features=500, bias=True)
- Sigmoid()
- Dropout(p=0.5)
- Linear(in_features=500, out_features=2, bias=True)

The best CNN-based architecture:

- Conv1d(1062, 400, kernel_size=(3,), stride=(1,), padding=(1,))
- ReLU()
- MaxPool1d(kernel_size=3, stride=1, padding=1, dilation=1, ceil_mode=False)
- Dropout(p=0.5)
- Linear(in_features=400, out_features=100, bias=True)
- Sigmoid()
- Dropout(p=0.5)
- Linear(in_features=100, out_features=2, bias=True)

The best hybrid RNN-CNN-based architecture:

- Conv1d(1062, 800, kernel_size=(5,), stride=(1,), padding=(2,))
- ReLU()
- MaxPool1d(kernel_size=3, stride=1, padding=1, dilation=1, ceil_mode=False)
- Conv1d(800, 600, kernel_size=(5,), stride=(1,), padding=(2,))
- ReLU()
- MaxPool1d(kernel_size=3, stride=1, padding=1, dilation=1, ceil_mode=False)
- LSTM(600, 400, bidirectional=True)
- Dropout(p=0.5)
- Linear(in_features=800, out_features=200, bias=True)
- Sigmoid()
- Dropout(p=0.5)
- Linear(in_features=200, out_features=2, bias=True)
