# Supplementary material for: Deep learning approach for predicting functional Z-DNA regions using omics data
Source: Sci Rep. 2020 Nov 5;10:19134. doi: 10.1038/s41598-020-76203-1 (PMC7644757; doi:10.1038/s41598-020-76203-1)
Supplement: Supplementary file 12 — Supplementary Table S3. [file 41598_2020_76203_MOESM12_ESM.docx]

**Deep learning approach for predicting functional Z-DNA regions using omics data**

**Nazar Beknazarov, Seungmin Jin and Maria Poptsova**

**Supplementary Table S3.** List of features used in DeepZ model

**DNase-Seq**

DNase I hypersensitive sites

**RNA polymerase**

| 1 | POLR3GL | 5 | POLR2M | 9 | RNA polymerase II |
| --- | --- | --- | --- | --- | --- |
| 2 | POLR2A | 6 | POLR3A | 10 | RNA polymerase III |
| 3 | POLR2B | 7 | POLR3D |  |  |
| 4 | POLR2G | 8 | POLR3G |  |  |

**Histone marks**

| 1 | macroH2A2 | 35 | H2ac | 69 | H3K9K14ac |
| --- | --- | --- | --- | --- | --- |
| 2 | H1.0 | 36 | H3 | 70 | H3K9ac |
| 3 | H1.2 | 37 | H3.1 | 71 | H3K9me1 |
| 4 | H1.4 | 38 | H3.3 | 72 | H3K9me2 |
| 5 | H1.4K34ac | 39 | H3.3 K27M mutant | 73 | H3K9me3 |
| 6 | H1.X | 40 | H3.3B | 74 | H3R17me2 |
| 7 | H1B | 41 | H3.Y | 75 | H3R26cit |
| 8 | H1K85ac | 42 | H3K122ac | 76 | H3R2me2 |
| 9 | H1t | 43 | H3K14ac | 77 | H3R8me2 |
| 10 | H2 | 44 | H3K14me3 | 78 | H3T11ph |
| 11 | H2A | 45 | H3K18ac | 79 | H3T45ph |
| 12 | H2A.X | 46 | H3K18cr | 80 | H3ac |
| 13 | H2A.XS139ph | 47 | H3K23ac | 81 | H4 |
| 14 | H2A.Z | 48 | H3K27ac | 82 | H4K12ac |
| 15 | H2A.Z.1 | 49 | H3K27me1 | 83 | H4K16ac |
| 16 | H2A.Z.2 | 50 | H3K27me2 | 84 | H4K20me1 |
| 17 | H2A.ZK4K7K11ac | 51 | H3K27me3 | 85 | H4K20me2 |
| 18 | H2A.Zac | 52 | H3K36ac | 86 | H4K20me3 |
| 19 | H2AK119Ub | 53 | H3K36me1 | 87 | H4K4me3 |
| 20 | H2AK120Ub | 54 | H3K36me2 | 88 | H4K5K8K12K16ac |
| 21 | H2AK5ac | 55 | H3K36me3 | 89 | H4K5K8K16ac |
| 22 | H2AK9ac | 56 | H3K4ac | 90 | H4K5K8ac |
| 23 | H2AT120ph | 57 | H3K4me | 91 | H4K5ac |
| 24 | H2Aub | 58 | H3K4me1 | 92 | H4K8ac |
| 25 | H2B | 59 | H3K4me2 | 93 | H4K91ac |
| 26 | H2BK120ac | 60 | H3K4me3 | 94 | H4R3me2 |
| 27 | H2BK120ub | 61 | H3K4ox | 95 | H4ac |
| 28 | H2BK120ub1 | 62 | H3K56ac | 96 | Pan lysine acetylation |
| 29 | H2BK15ac | 63 | H3K5ac | 97 | Pan lysine crotonylation |
| 30 | H2BK20ac | 64 | H3K79me1 | 98 | WAPL |
| 31 | H2BK5ac | 65 | H3K79me2 | 99 | macroH2A1 |
| 32 | H2BP1me1 | 66 | H3K79me3 | 100 | macroH2A1.2 |
| 33 | H2BS112GlcNAc | 67 | H3K79suc |  |  |
| 34 | H2Bub | 68 | H3K9K14K18K23K27ac |  |  |

**Transcription Factors and others**

| 1 | 5-hmC | 316 | HINFP | 631 | PML |
| --- | --- | --- | --- | --- | --- |
| 2 | 5-mC | 317 | HIRA | 632 | POU2AF1 |
| 3 | 8-Hydroxydeoxyguanosine | 318 | HIV Tat | 633 | POU2F1 |
| 4 | AATF | 319 | HIVEP3 | 634 | POU2F2 |
| 5 | ADNP2 | 320 | HJURP | 635 | POU2F3 |
| 6 | AFF1 | 321 | HMBOX1 | 636 | POU3F2 |
| 7 | AFF4 | 322 | HMGA1 | 637 | POU5F1 |
| 8 | AGO1 | 323 | HMGB1 | 638 | PPARA |
| 9 | AGO2 | 324 | HMGB2 | 639 | PPARG |
| 10 | AHCTF1 | 325 | HMGN1 | 640 | PPARGC1A |
| 11 | AHR | 326 | HMGN3 | 641 | PRAME |
| 12 | AHRR | 327 | HNF1A | 642 | PRDM1 |
| 13 | ALKBH1 | 328 | HNF1B | 643 | PRDM11 |
| 14 | ALKBH3 | 329 | HNF4A | 644 | PRDM14 |
| 15 | ALYREF | 330 | HNF4G | 645 | PRDM4 |
| 16 | AML1-ETO | 331 | HNRNPC | 646 | PRKCQ |
| 17 | APC | 332 | HNRNPH1 | 647 | PRKDC |
| 18 | APOBEC3B | 333 | HNRNPK | 648 | PRMT1 |
| 19 | AR | 334 | HNRNPL | 649 | PRMT5 |
| 20 | ARID1A | 335 | HNRNPLL | 650 | PROX1 |
| 21 | ARID1B | 336 | HNRNPUL1 | 651 | PRPF4 |
| 22 | ARID2 | 337 | HOXA11 | 652 | PSIP1 |
| 23 | ARID3A | 338 | HOXA13 | 653 | PSMB5 |
| 24 | ARID3B | 339 | HOXA4 | 654 | PSMD1 |
| 25 | ARID5B | 340 | HOXA5 | 655 | PTBP1 |
| 26 | ARNT | 341 | HOXA6 | 656 | PTEN |
| 27 | ARNT2 | 342 | HOXA9 | 657 | PYGO2 |
| 28 | ARNTL | 343 | HOXB1 | 658 | Pan-acetyllysine |
| 29 | ARNTL2 | 344 | HOXB13 | 659 | RAD21 |
| 30 | ARRB1 | 345 | HOXB2 | 660 | RAD51 |
| 31 | ASCL1 | 346 | HOXB4 | 661 | RAG1 |
| 32 | ASF1A | 347 | HOXB5 | 662 | RAG2 |
| 33 | ASH1L | 348 | HOXB6 | 663 | RARA |
| 34 | ASH2L | 349 | HOXB7 | 664 | RARB |
| 35 | ASUN | 350 | HOXB8 | 665 | RB1 |
| 36 | ASXL1 | 351 | HOXC11 | 666 | RBBP5 |
| 37 | ASXL2 | 352 | HOXC13 | 667 | RBBP7 |
| 38 | ATF1 | 353 | HOXC5 | 668 | RBFOX2 |
| 39 | ATF2 | 354 | HOXC8 | 669 | RBL1 |
| 40 | ATF3 | 355 | HOXC9 | 670 | RBL2 |
| 41 | ATF4 | 356 | HOXD1 | 671 | RBM22 |
| 42 | ATF6 | 357 | HOXD11 | 672 | RBM25 |
| 43 | ATF7 | 358 | HOXD13 | 673 | RBM39 |
| 44 | ATF7IP | 359 | HOXD4 | 674 | RBP1 |
| 45 | ATR | 360 | HOXD9 | 675 | RBPJ |
| 46 | ATRX | 361 | HSF1 | 676 | RCOR1 |
| 47 | BACH1 | 362 | Hepatitis B Virus X antigen | 677 | REL |
| 48 | BACH2 | 363 | ICE1 | 678 | RELA |
| 49 | BANP | 364 | ICE2 | 679 | RELB |
| 50 | BAP1 | 365 | ID1 | 680 | REST |
| 51 | BARX1 | 366 | ID2 | 681 | RFX2 |
| 52 | BATF | 367 | ID3 | 682 | RFX5 |
| 53 | BATF3 | 368 | ID4 | 683 | RFX7 |
| 54 | BBX | 369 | IGF1 | 684 | RING1 |
| 55 | BCL10 | 370 | IKZF1 | 685 | RLF |
| 56 | BCL11A | 371 | IKZF5 | 686 | RNF2 |
| 57 | BCL11B | 372 | ILF3 | 687 | RORC |
| 58 | BCL3 | 373 | ING2 | 688 | RPA1 |
| 59 | BCL6 | 374 | ING5 | 689 | RPA2 |
| 60 | BCLAF1 | 375 | INO80 | 690 | RTA |
| 61 | BCOR | 376 | INSR | 691 | RUNX1 |
| 62 | BDP1 | 377 | INTS12 | 692 | RUNX1T1 |
| 63 | BHLHE40 | 378 | INTS13 | 693 | RUNX2 |
| 64 | BHLHE41 | 379 | INTS3 | 694 | RUNX3 |
| 65 | BMI | 380 | IRF1 | 695 | RUVBL1 |
| 66 | BMI1 | 381 | IRF2 | 696 | RUVBL2 |
| 67 | BPTF | 382 | IRF3 | 697 | RXRA |
| 68 | BRAF | 383 | IRF4 | 698 | RYBP |
| 69 | BRCA1 | 384 | IRF5 | 699 | SAFB |
| 70 | BRCA2 | 385 | IRF8 | 700 | SAFB2 |
| 71 | BRD1 | 386 | IRF9 | 701 | SAG |
| 72 | BRD2 | 387 | IRX2 | 702 | SALL3 |
| 73 | BRD3 | 388 | IRX5 | 703 | SALL4 |
| 74 | BRD4 | 389 | ISL1 | 704 | SAP30 |
| 75 | BRD7 | 390 | ISL2 | 705 | SATB1 |
| 76 | BRD9 | 391 | JARID2 | 706 | SCML2 |
| 77 | BRF1 | 392 | JAZF1 | 707 | SETD1A |
| 78 | BRF2 | 393 | JDP2 | 708 | SETDB1 |
| 79 | BRPF3 | 394 | JMJD1C | 709 | SETX |
| 80 | Biotin | 395 | JMJD6 | 710 | SF3B1 |
| 81 | BrdU | 396 | JUN | 711 | SFMBT1 |
| 82 | C10orf12 | 397 | JUNB | 712 | SFPQ |
| 83 | C17orf49 | 398 | JUND | 713 | SIN3A |
| 84 | C17orf96 | 399 | KAT2A | 714 | SIPA1 |
| 85 | CAMTA1 | 400 | KAT2B | 715 | SIRT1 |
| 86 | CARM1 | 401 | KAT5 | 716 | SIRT3 |
| 87 | CASP8AP2 | 402 | KAT7 | 717 | SIRT6 |
| 88 | CBFA2T2 | 403 | KAT8 | 718 | SIRT7 |
| 89 | CBFA2T3 | 404 | KCMF1 | 719 | SIX1 |
| 90 | CBFB | 405 | KDM1A | 720 | SIX2 |
| 91 | CBX1 | 406 | KDM2B | 721 | SIX5 |
| 92 | CBX2 | 407 | KDM3A | 722 | SKI |
| 93 | CBX3 | 408 | KDM3B | 723 | SKP2 |
| 94 | CBX4 | 409 | KDM4A | 724 | SLBP |
| 95 | CBX5 | 410 | KDM4C | 725 | SMAD1 |
| 96 | CBX6 | 411 | KDM5A | 726 | SMAD2 |
| 97 | CBX7 | 412 | KDM5B | 727 | SMAD3 |
| 98 | CBX8 | 413 | KDM5C | 728 | SMAD4 |
| 99 | CCAR2 | 414 | KDM5D | 729 | SMAD5 |
| 100 | CCDC101 | 415 | KDM6A | 730 | SMARCA1 |
| 101 | CCND1 | 416 | KDM6B | 731 | SMARCA2 |
| 102 | CCND2 | 417 | KLF1 | 732 | SMARCA4 |
| 103 | CCNT1 | 418 | KLF11 | 733 | SMARCA5 |
| 104 | CCNT2 | 419 | KLF13 | 734 | SMARCB1 |
| 105 | CD74 | 420 | KLF15 | 735 | SMARCC1 |
| 106 | CDC5L | 421 | KLF4 | 736 | SMARCC2 |
| 107 | CDC73 | 422 | KLF5 | 737 | SMARCE1 |
| 108 | CDCA5 | 423 | KLF6 | 738 | SMC1A |
| 109 | CDK12 | 424 | KMT2A | 739 | SMC3 |
| 110 | CDK2 | 425 | KMT2B | 740 | SMC4 |
| 111 | CDK6 | 426 | KMT2C | 741 | SMCHD1 |
| 112 | CDK7 | 427 | KMT2D | 742 | SMN1 |
| 113 | CDK8 | 428 | KSHV LANA | 743 | SNAI2 |
| 114 | CDK9 | 429 | L3MBTL2 | 744 | SNAPC1 |
| 115 | CDKN1B | 430 | L3MBTL3 | 745 | SNAPC2 |
| 116 | CDX1 | 431 | LARP7 | 746 | SNAPC4 |
| 117 | CDX2 | 432 | LCOR | 747 | SNAPC5 |
| 118 | CEBPA | 433 | LCORL | 748 | SNRNP70 |
| 119 | CEBPB | 434 | LDB1 | 749 | SOD1 |
| 120 | CEBPD | 435 | LEF1 | 750 | SON |
| 121 | CEBPZ | 436 | LEO1 | 751 | SOX10 |
| 122 | CHAF1A | 437 | LHX2 | 752 | SOX11 |
| 123 | CHAT | 438 | LHX4 | 753 | SOX17 |
| 124 | CHD1 | 439 | LHX5 | 754 | SOX2 |
| 125 | CHD2 | 440 | LHX6 | 755 | SOX3 |
| 126 | CHD3 | 441 | LIG4 | 756 | SOX8 |
| 127 | CHD4 | 442 | LIN9 | 757 | SOX9 |
| 128 | CHD7 | 443 | LMNA | 758 | SP1 |
| 129 | CHD8 | 444 | LMNB1 | 759 | SP140 |
| 130 | CIC | 445 | LMO1 | 760 | SP2 |
| 131 | CIITA | 446 | LMO2 | 761 | SP4 |
| 132 | CLOCK | 447 | LMO3 | 762 | SPDEF |
| 133 | CNOT3 | 448 | LMTK3 | 763 | SPI1 |
| 134 | COBLL1 | 449 | LRIF1 | 764 | SPIN1 |
| 135 | CPSF3 | 450 | LRWD1 | 765 | SRCAP |
| 136 | CPSF3L | 451 | LYL1 | 766 | SREBF1 |
| 137 | CREB1 | 452 | MAF | 767 | SREBF2 |
| 138 | CREB3L1 | 453 | MAF1 | 768 | SRF |
| 139 | CREBBP | 454 | MAFB | 769 | SRPK1 |
| 140 | CREBL2 | 455 | MAFF | 770 | SRPK2 |
| 141 | CRTC2 | 456 | MAFG | 771 | SRSF1 |
| 142 | CRY1 | 457 | MAFK | 772 | SRSF3 |
| 143 | CSF1R | 458 | MAML1 | 773 | SRSF4 |
| 144 | CSNK2A1 | 459 | MAML3 | 774 | SRSF7 |
| 145 | CSTF3 | 460 | MAP1LC3B | 775 | SRSF9 |
| 146 | CTBP1 | 461 | MAP2K1 | 776 | SS18 |
| 147 | CTBP2 | 462 | MAPK1 | 777 | SSRP1 |
| 148 | CTCF | 463 | MAPK14 | 778 | STAG1 |
| 149 | CTCFL | 464 | MAX | 779 | STAG2 |
| 150 | CTNNB1 | 465 | MAZ | 780 | STAT1 |
| 151 | CTR9 | 466 | MBD2 | 781 | STAT2 |
| 152 | CUL4A | 467 | MBD3 | 782 | STAT3 |
| 153 | CUX1 | 468 | MBD3L2 | 783 | STAT4 |
| 154 | CXXC1 | 469 | MBD4 | 784 | STAT5 |
| 155 | Cas9 | 470 | MCM2 | 785 | STAT5A |
| 156 | Cyclobutane pyrimidine dimers | 471 | MCM3 | 786 | STAT5B |
| 157 | DAXX | 472 | MCPV ST | 787 | STAT6 |
| 158 | DCP1A | 473 | MCRS1 | 788 | SUMO1 |
| 159 | DDIT3 | 474 | MDM2 | 789 | SUMO2 |
| 160 | DDX11 | 475 | MECOM | 790 | SUPT20H |
| 161 | DDX5 | 476 | MECP2 | 791 | SUPT5H |
| 162 | DEAF1 | 477 | MED1 | 792 | SUPT6H |
| 163 | DEK | 478 | MED12 | 793 | SUZ12 |
| 164 | DICER1 | 479 | MED25 | 794 | SVIL |
| 165 | DMC1 | 480 | MED26 | 795 | SVS-1 |
| 166 | DMRT2 | 481 | MEF2A | 796 | T |
| 167 | DNA2 | 482 | MEF2B | 797 | TAF1 |
| 168 | DNMT1 | 483 | MEF2C | 798 | TAF15 |
| 169 | DNMT3A | 484 | MEIS1 | 799 | TAF2 |
| 170 | DNMT3B | 485 | MEN1 | 800 | TAF3 |
| 171 | DOT1L | 486 | MEOX2 | 801 | TAF7 |
| 172 | DPF2 | 487 | MEPCE | 802 | TAL1 |
| 173 | DPPA3 | 488 | METTL14 | 803 | TARDBP |
| 174 | DR1 | 489 | METTL3 | 804 | TAZ |
| 175 | DRAP1 | 490 | MGEA5 | 805 | TBL1XR1 |
| 176 | DTL | 491 | MIF | 806 | TBL1Y |
| 177 | DUX4 | 492 | MITF | 807 | TBP |
| 178 | DYRK1A | 493 | MIXL1 | 808 | TBX2 |
| 179 | E2F1 | 494 | MLL-AF4 | 809 | TBX21 |
| 180 | E2F2 | 495 | MLLT1 | 810 | TBX5 |
| 181 | E2F3 | 496 | MLLT3 | 811 | TCEA2 |
| 182 | E2F4 | 497 | MLXIP | 812 | TCF12 |
| 183 | E2F6 | 498 | MORC2 | 813 | TCF21 |
| 184 | E2F7 | 499 | MPHOSPH8 | 814 | TCF3 |
| 185 | E2F8 | 500 | MRE11A | 815 | TCF4 |
| 186 | EBF1 | 501 | MSC | 816 | TCF7L1 |
| 187 | EBF3 | 502 | MTA2 | 817 | TCF7L2 |
| 188 | EBNA1 | 503 | MTA3 | 818 | TCFL5 |
| 189 | EBNA2 | 504 | MTF1 | 819 | TDRD3 |
| 190 | EBNA3 | 505 | MTHFD1 | 820 | TEAD1 |
| 191 | EBV-ZEBRA | 506 | MTOR | 821 | TEAD4 |
| 192 | EED | 507 | MXD3 | 822 | TERF1 |
| 193 | EGFR | 508 | MXI1 | 823 | TERF2 |
| 194 | EGR1 | 509 | MYB | 824 | TET1 |
| 195 | EGR2 | 510 | MYBL1 | 825 | TET2 |
| 196 | EHF | 511 | MYBL2 | 826 | TET3 |
| 197 | EHMT2 | 512 | MYC | 827 | TFAM |
| 198 | ELF1 | 513 | MYCN | 828 | TFAP2A |
| 199 | ELF2 | 514 | MYF5 | 829 | TFAP2C |
| 200 | ELF3 | 515 | MYF6 | 830 | TFAP4 |
| 201 | ELF4 | 516 | MYH11 | 831 | TFCP2 |
| 202 | ELK1 | 517 | MYNN | 832 | TFDP1 |
| 203 | ELK3 | 518 | MYOCD | 833 | TFEB |
| 204 | ELK4 | 519 | MYOD1 | 834 | THAP1 |
| 205 | ELL | 520 | MYOG | 835 | THAP11 |
| 206 | ELL2 | 521 | MethylCap | 836 | THAP4 |
| 207 | EOMES | 522 | NAB2 | 837 | TLE3 |
| 208 | EP300 | 523 | NANOG | 838 | TLX1 |
| 209 | EP400 | 524 | NCAPG | 839 | TOE1 |
| 210 | EPAS1 | 525 | NCAPG2 | 840 | TOP1 |
| 211 | EPCAM | 526 | NCAPH2 | 841 | TOP2A |
| 212 | ERCC2 | 527 | NCOA1 | 842 | TOP2B |
| 213 | ERCC3 | 528 | NCOA2 | 843 | TP53 |
| 214 | ERCC6 | 529 | NCOA3 | 844 | TP53BP1 |
| 215 | ERCC8 | 530 | NCOA4 | 845 | TP63 |
| 216 | ERG | 531 | NCOR1 | 846 | TP73 |
| 217 | ESR1 | 532 | NCOR2 | 847 | TRF2 |
| 218 | ESR2 | 533 | NELFA | 848 | TRIM24 |
| 219 | ESRRA | 534 | NELFE | 849 | TRIM25 |
| 220 | ETS1 | 535 | NETO2 | 850 | TRIM28 |
| 221 | ETS2 | 536 | NEUROD1 | 851 | TRP47 |
| 222 | ETV1 | 537 | NEUROD2 | 852 | TRPS1 |
| 223 | ETV4 | 538 | NFAT5 | 853 | TRRAP |
| 224 | ETV5 | 539 | NFATC1 | 854 | TSC1 |
| 225 | ETV6 | 540 | NFATC2 | 855 | TSHZ1 |
| 226 | EVX1 | 541 | NFE2 | 856 | TTF2 |
| 227 | EWSR1 | 542 | NFE2L1 | 857 | TWIST1 |
| 228 | EZH1 | 543 | NFE2L2 | 858 | U2AF1 |
| 229 | EZH2 | 544 | NFE2L3 | 859 | U2AF2 |
| 230 | Epitope tags | 545 | NFIB | 860 | UBE2I |
| 231 | F2RL1 | 546 | NFIC | 861 | UBE3A |
| 232 | FAM208A | 547 | NFKB1 | 862 | UBP1 |
| 233 | FANCD2 | 548 | NFKB2 | 863 | UBTF |
| 234 | FERD3L | 549 | NFKBIA | 864 | USF1 |
| 235 | FGFR1 | 550 | NFYA | 865 | USF2 |
| 236 | FIP1L1 | 551 | NFYB | 866 | USP7 |
| 237 | FLI1 | 552 | NFYC | 867 | VDR |
| 238 | FOS | 553 | NHLH1 | 868 | VEZF1 |
| 239 | FOSB | 554 | NIPBL | 869 | VRK1 |
| 240 | FOSL1 | 555 | NKX2-1 | 870 | VSV-G |
| 241 | FOSL2 | 556 | NKX2-3 | 871 | WDHD1 |
| 242 | FOXA1 | 557 | NKX2-5 | 872 | WDR5 |
| 243 | FOXA2 | 558 | NKX2-8 | 873 | WHSC1 |
| 244 | FOXC1 | 559 | NKX3-1 | 874 | WIZ |
| 245 | FOXD3 | 560 | NKX6-1 | 875 | WRN |
| 246 | FOXF1 | 561 | NLRP2 | 876 | WRNIP1 |
| 247 | FOXF2 | 562 | NME2 | 877 | WWTR1 |
| 248 | FOXH1 | 563 | NONO | 878 | XBP1 |
| 249 | FOXJ2 | 564 | NOS2 | 879 | XRCC4 |
| 250 | FOXJ3 | 565 | NOTCH1 | 880 | XRCC5 |
| 251 | FOXK1 | 566 | NOTCH3 | 881 | XRN2 |
| 252 | FOXM1 | 567 | NPAT | 882 | YAP1 |
| 253 | FOXN3 | 568 | NR1D1 | 883 | YBX1 |
| 254 | FOXO1 | 569 | NR1H2 | 884 | YEATS4 |
| 255 | FOXO4 | 570 | NR1H3 | 885 | YY1 |
| 256 | FOXP1 | 571 | NR1H4 | 886 | YY2 |
| 257 | FOXP2 | 572 | NR1I2 | 887 | ZBTB10 |
| 258 | FOXP3 | 573 | NR2C2 | 888 | ZBTB16 |
| 259 | FOXQ1 | 574 | NR2E3 | 889 | ZBTB17 |
| 260 | FOXR2 | 575 | NR2F1 | 890 | ZBTB2 |
| 261 | FUS | 576 | NR2F2 | 891 | ZBTB24 |
| 262 | FXR1 | 577 | NR2F6 | 892 | ZBTB33 |
| 263 | FXR2 | 578 | NR3C1 | 893 | ZBTB39 |
| 264 | GABPA | 579 | NR3C2 | 894 | ZBTB4 |
| 265 | GATA1 | 580 | NR4A1 | 895 | ZBTB44 |
| 266 | GATA2 | 581 | NR4A2 | 896 | ZBTB48 |
| 267 | GATA3 | 582 | NR5A1 | 897 | ZBTB7A |
| 268 | GATA4 | 583 | NR5A2 | 898 | ZC3H11A |
| 269 | GATA6 | 584 | NRF1 | 899 | ZC3H8 |
| 270 | GATAD1 | 585 | NUP153 | 900 | ZEB1 |
| 271 | GATAD2A | 586 | NUP98 | 901 | ZEB2 |
| 272 | GATAD2B | 587 | NUTM1 | 902 | ZFHX4 |
| 273 | GFI1 | 588 | NXF1 | 903 | ZFP42 |
| 274 | GFI1B | 589 | O-GlcNAc | 904 | ZFP64 |
| 275 | GFP | 590 | OGT | 905 | ZFX |
| 276 | GLI2 | 591 | ONECUT2 | 906 | ZHX2 |
| 277 | GLTSCR1 | 592 | ORC1 | 907 | ZIC2 |
| 278 | GLYR1 | 593 | ORC2 | 908 | ZKSCAN1 |
| 279 | GMEB2 | 594 | OTX2 | 909 | ZMIZ1 |
| 280 | GPS2 | 595 | OVOL2 | 910 | ZMYM2 |
| 281 | GREB1 | 596 | PADI2 | 911 | ZMYND11 |
| 282 | GRHL1 | 597 | PAF1 | 912 | ZMYND8 |
| 283 | GRHL2 | 598 | PALB2 | 913 | ZNF12 |
| 284 | GRHL3 | 599 | PARK7 | 914 | ZNF143 |
| 285 | GTF2B | 600 | PARP1 | 915 | ZNF165 |
| 286 | GTF2F1 | 601 | PAX5 | 916 | ZNF207 |
| 287 | GTF2I | 602 | PAX6 | 917 | ZNF217 |
| 288 | GTF3C5 | 603 | PAX7 | 918 | ZNF263 |
| 289 | GUCY1B3 | 604 | PAX8 | 919 | ZNF266 |
| 290 | GZF1 | 605 | PBX1 | 920 | ZNF274 |
| 291 | H3K9K14K18K23K27ac | 606 | PBX2 | 921 | ZNF280D |
| 292 | HAND1 | 607 | PBX3 | 922 | ZNF281 |
| 293 | HAND2 | 608 | PBX4 | 923 | ZNF3 |
| 294 | HAT1 | 609 | PBXIP1 | 924 | ZNF341 |
| 295 | HBZ | 610 | PCBP1 | 925 | ZNF366 |
| 296 | HCFC1 | 611 | PCBP2 | 926 | ZNF384 |
| 297 | HDAC1 | 612 | PCF11 | 927 | ZNF395 |
| 298 | HDAC2 | 613 | PCGF1 | 928 | ZNF486 |
| 299 | HDAC3 | 614 | PCGF2 | 929 | ZNF516 |
| 300 | HDAC4 | 615 | PCGF5 | 930 | ZNF532 |
| 301 | HDAC6 | 616 | PCGF6 | 931 | ZNF581 |
| 302 | HDAC8 | 617 | PDX1 | 932 | ZNF639 |
| 303 | HECTD1 | 618 | PGBD5 | 933 | ZNF644 |
| 304 | HES4 | 619 | PGR | 934 | ZNF711 |
| 305 | HES5 | 620 | PHC1 | 935 | ZNF750 |
| 306 | HES7 | 621 | PHF2 | 936 | ZNF76 |
| 307 | HESX1 | 622 | PHF6 | 937 | ZNF768 |
| 308 | HEXIM1 | 623 | PHF8 | 938 | ZNF83 |
| 309 | HEY1 | 624 | PHIP | 939 | ZNF84 |
| 310 | HEY2 | 625 | PHOX2B | 940 | ZNF92 |
| 311 | HEYL | 626 | PIAS1 | 941 | ZSCAN5A |
| 312 | HHEX | 627 | PIAS4 | 942 | ZSCAN5B |
| 313 | HIC1 | 628 | PITX1 | 943 | ZSCAN5D |
| 314 | HIC2 | 629 | PITX3 | 944 | ZZZ3 |
| 315 | HIF1A | 630 | PLAG1 | 945 | pFM2 |
|  |  |  |  | 946 | GTF3C1 |

Methylation sites

| 1 | 5fc 1st-Pb | 6 | 5fc ICM | 11 | 5fc hESC |
| --- | --- | --- | --- | --- | --- |
| 2 | 5fc 2cell | 7 | 5fc Morula | 12 | 5fc malePN |
| 3 | 5fc 4cell | 8 | 5fc Oocyte | 13 | 8-Oxoguanine |
| 4 | 5fc 8cell | 9 | 5fc Sperm |  |  |
| 5 | 5fc TE | 10 | 5fc femalePN |  |  |

Z-HUNT features

| 1 | B-Z Transition (A-S) | 2 | B-Z Transition (S-A) | 3 | Z-Z Transition (A-S) | 4 | Z-Z Transition (S-A) |
| --- | --- | --- | --- | --- | --- | --- | --- |
